# Zinc transporters and their functional integration in mammalian cells

**DOI:** 10.1016/j.jbc.2021.100320

**Published:** 2021-01-22

**Authors:** Taiho Kambe, Kathryn M. Taylor, Dax Fu

**Affiliations:** 1 Division of Integrated Life Science, Graduate School of Biostudies, Kyoto University, Kyoto, Japan; 2 School of Pharmacy and Pharmaceutical Sciences, Cardiff University, Cardiff, United Kingdom; 3 Department of Physiology, Johns Hopkins School of Medicine, Johns Hopkins University, Baltimore, Maryland, USA

**Keywords:** zinc, transport metal, transporter, signal transduction, endoplasmic reticulum stress (ER stress), enzyme processing, protein–protein interaction, proteomics, CDF, cation diffusion facilitator, CTD, C-terminal domain, ECD, extracellular domain, ECM, extracellular matrix, ER, endoplasmic reticulum, ICP-MS, inductively coupled plasma–mass spectrometry, LOF, loss-of-function, SCD-EDS, spondylocheiro dysplastic form of Ehlers–Danlos syndrome, SNP, single nucleotide polymorphism, SOD, superoxide dismutase, TMD, transmembrane domain, ZnT, zinc transporter

## Abstract

Zinc is a ubiquitous biological metal in all living organisms. The spatiotemporal zinc dynamics in cells provide crucial cellular signaling opportunities, but also challenges for intracellular zinc homeostasis with broad disease implications. Zinc transporters play a central role in regulating cellular zinc balance and subcellular zinc distributions. The discoveries of two complementary families of mammalian zinc transporters (ZnTs and ZIPs) in the mid-1990s spurred much speculation on their metal selectivity and cellular functions. After two decades of research, we have arrived at a biochemical description of zinc transport. However, *in vitro* functions are fundamentally different from those in living cells, where mammalian zinc transporters are directed to specific subcellular locations, engaged in dedicated macromolecular machineries, and connected with diverse cellular processes. Hence, the molecular functions of individual zinc transporters are reshaped and deeply integrated in cells to promote the utilization of zinc chemistry to perform enzymatic reactions, tune cellular responsiveness to pathophysiologic signals, and safeguard cellular homeostasis. At present, the underlying mechanisms driving the functional integration of mammalian zinc transporters are largely unknown. This knowledge gap has motivated a shift of the research focus from *in vitro* studies of purified zinc transporters to *in cell* studies of mammalian zinc transporters in the context of their subcellular locations and protein interactions. In this review, we will outline how knowledge of zinc transporters has been accumulated from *in-test-tube* to *in-cell* studies, highlighting new insights and paradigm shifts in our understanding of the molecular and cellular basis of mammalian zinc transporter functions.

Zinc is a defining feature of eukaryotic proteomes, populating in zinc-binding proteins that are encoded by ∼10% of the human genome (1, 2). While zinc is fundamental to eukaryotic cellular biology, it is a later addition to proteomes after the advent of a geochemical shift of an ancient high-sulfide ocean to the modern oxidizing, sulfate-rich one (3, 4). The release of the sulfide-bound zinc provided zinc bioavailability to prompt a burst in the innovation of protein structures such as zinc fingers with consequences for quickening the rise and diversification of eukaryotes in evolution (5). However, zinc utilization in eukaryotes came at the expense of increasing risk for interference with preexisting cellular machineries evolved earlier. Accordingly, the human body harnesses potentially toxic zinc chemistry in a spatially confined manner to avoid cytotoxicity. At the organ level, zinc is highly enriched in the hippocampus and neocortex region of the brain, prostate gland, and islets of Langerhans of the pancreas (6). These tissues accumulate abundant zinc into secretory pathways and release the stored zinc on demand. At the cellular level, the total cellular zinc content is submillimolar with uneven distributions ranging from a picomolar range of free cytosolic zinc to over 20 mM in specialized secretory vesicles (7, 8, 9, 10, 11). For example, zinc is highly enriched in pancreatic β-cells for insulin packaging, prostate epithelial cells for citrate production, and mammary epithelial cells for lactation (12). At the protein level, zinc transporters selectively capture and transport zinc ions across the membrane barriers to control zinc gradients across various biological membranes.

Mammalian zinc transporters belong to two complementary protein families (13): the Zinc Transporter (ZnT) (14) and Zrt, Irt-like Protein (ZIP) (15). Ten ZnT and 14 ZIP homologs are encoded by *SLC30A1-10* and *SLC39A1-14* genes in humans, respectively (13). Common to all zinc transporters is the ability of selective zinc binding, but bound zinc ions in ZnTs and ZIPs are transported in opposite directions. ZnTs are efflux transporters responsible for removing excess zinc in the cytoplasm, whereas ZIPs are uptake transporters that replenish cytosolic zinc (Fig. 1, *A*–*C*). The complementary functions of ZnTs and ZIPs stabilize the cytosolic zinc concentration around a homeostatic setpoint while enriching zinc in the lumen of specific subcellular compartments to support zinc-dependent cellular processes.

**Figure 1:**
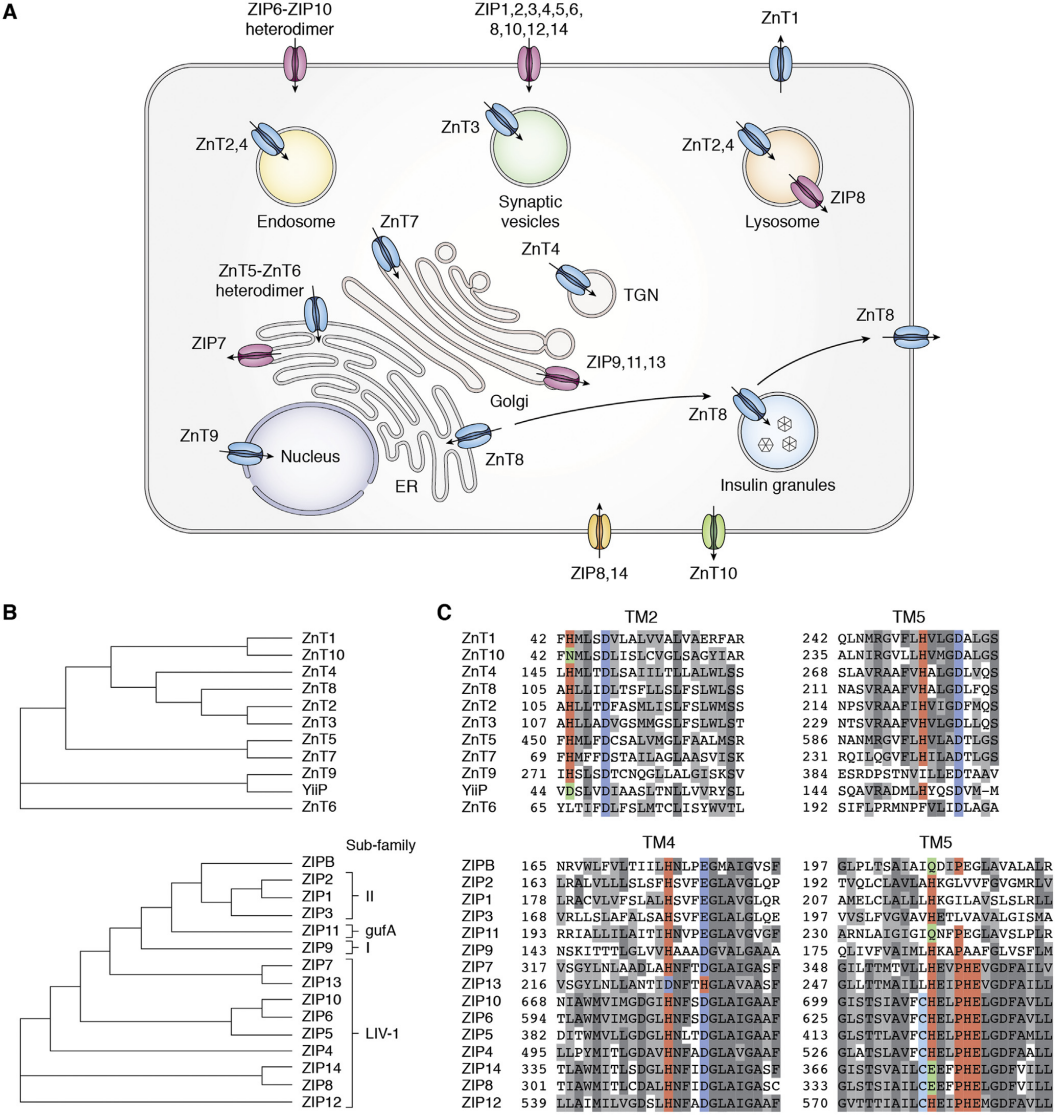
**Two complementary families of mammalian zinc transporters**. *A*, subcellular localization of ZIP (*light pink*) and ZnT (*light blue*) that are generally thought to be zinc-specific transporters. ZIP8 and ZIP14 (*gold*) and ZnT10 (*pale green*) transport manganese in addition to zinc. *B*, phylogenetic trees of ZnT (*upper*) and ZIP (*lower*). *C*, sequence alignments of key coordinating residues in the transport sites in bacterial YiiP (*upper*) and ZIPB (*lower*). *Light coral shading* highlights conserved His residues. *Light blue shading* highlights conserved Asp and Glu residues. His and Asp residues are reversed in TM4 of ZIP13. *Pale turquoise shading* highlights the conserved Cys residue in cell surface located LIV-1 family members of ZIPs that have been postulated to bind to the N-terminal CPALLY motif. *Light green shading* highlights loss of the conserved His, which in the case of ZnT10, ZIP8, and ZIP14 results in loss of zinc specificity.

The multisite localizations of zinc transporters in mammalian cells increase the number of protein–protein interactions that drive functional cross talks between zinc transporters and an array of cellular machineries performing diverse cellular processes beyond zinc transport. Hence, mammalian zinc transporters are organized in a functional hierarchy across scales—from the fundamental chemistry of protein structures through subcellular localizations and macromolecular complexes up to cellular processes that constitute the molecular physiology of mammalian cells. Accordingly, this review will begin with *in-test-tube* studies of isolated zinc transporters to illuminate how zinc coordination and geometric arrangements of binding residues confer zinc selectivity in protein structures and how zinc bindings drive protein structural dynamics to move up or down zinc concentration gradients across the membrane. *In-cell* studies of zinc transporters further showcase how zinc transporters are directed to distinct subcellular locations and engaged in different protein complexes in response to environmental stimuli. The next level of functional integrations takes place in zinc-dependent biological processes in which individual zinc transporters coordinate networks of protein–protein interactions involved in cellular zinc signaling, endoplasmic reticulum (ER) homeostasis and unfolded protein response, and activation of zinc ectoenzymes in the early secretory pathway. The involvement of mammalian zinc transporters in diverse cellular processes provides insights into their global functions in human pathophysiology. Finally, we will summarize existing knowledge of loss-of-function (LOF) mutations and polymorphisms in human ZnTs and ZIPs and their clinical manifestations in major human diseases.

## Zinc selectivity and its structural basis

Cells acquire a variety of transition elements including iron, zinc, copper, manganese, cobalt, nickel, molybdenum, tungsten, chromium, and vanadium. These biological metals may exist in tightly bound forms such as metal-bound cofactors and proteins or nucleic-acid-bound species, or loosely bound forms in association with a diverse heterogeneous buffer. Biomolecules in the intracellular milieu have an extraordinary metal chelation capacity, probably containing an excess of high-affinity binding sites relative to the number of transition metal ions in cells (16). According to the Irving–Williams series for the binding stability of divalent metal ions (17), zinc and copper would form the most stable complexes, preferentially accumulating in complexes with biomolecules if metal ions are presented in equal amounts in the test tube. However, in living organisms, metal ions are selectively acquired in cells, where metal transporters override the intrinsic thermodynamic propensity by the selection and compartmentalization of metal ions to enable metal-specific cellular processes. As such, the metal selectivity of zinc transporters is an essential aspect of cell biology, regulating the composition of the intracellular metallome and safeguarding the fidelity of zinc delivery to the right subcellular compartments in the right amount. While ZnTs and ZIPs transport zinc ions in opposite directions by different transport mechanisms, common to these zinc transporters is an overall metal selectivity for zinc. This raises the question as to how different zinc transporters exploit zinc coordination chemistry to select zinc against other similar metal ions.

Zinc has an unusual electron configuration of [Ar]3d^10^. The completely filled d-orbital renders zinc redox-inert, and its ionic form has a fixed valence state of +2. Zinc is considered as a borderline soft-hard metal, being coordinated by both the sulfur atom of cysteine and nitrogen atom of histidine (soft base ligands) or by carboxylate oxygen atoms of aspartate and glutamate (hard base ligands) (18). Often, water molecules participate in zinc coordination and stabilize zinc ligation in particular conformations. In some cases, the binding of a water molecule to a positively charged zinc center reduces the pKa of water from 15.7 to ∼7, generating a hydroxide ion as a catalytically active species (19). A zinc ion with its filled d subshell has a marked preference for tetrahedral coordination geometry (20) dictated by the 18-electron rule (21). In addition, penta- and hexa-coordinated Zn(II) are frequently found in metalloenzymes with bound inhibitors or solvent molecules (20). The coordination geometry of each zinc binding site is broadly categorized by coordination number (N) and bond angles into tetrahedral (N = 4), trigonal bipyramidal (N = 5), and octahedral geometries (N = 6). Despite the variability in zinc coordination environments, the affinity toward Zn(II) is usually high in the μM to pM range in diverse zinc metalloproteins (22, 23).

**Figure 2:**
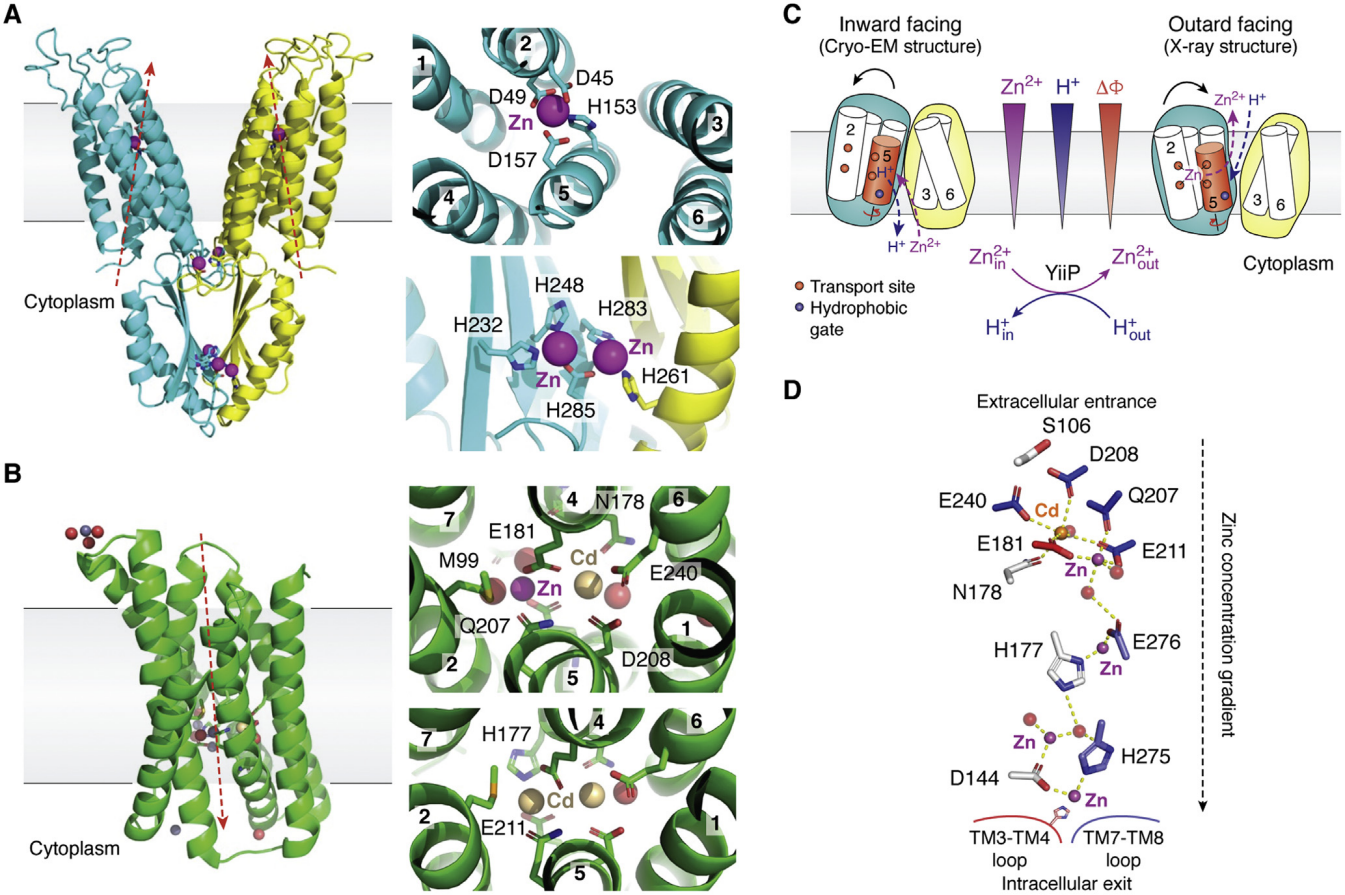
**Structural basis for zinc selectivity and mobility**. *A*, crystal structure of YiiP and close-up views of zinc binding sites in TMD (*upper*) and at the CTD interface (*lower*). *Red arrows* indicate directions of zinc transport. TMs and coordinating residues are labeled. *B*, crystal structure of ZIPB and close-up views of the binuclear metal center with a Zn and Cd ion (*upper*) and two Cd ions (*lower*). *C*, schematic model depicting zinc-for-proton exchange in YiiP. ΔΦ denotes the membrane potential. The tetrahedral transport site in YiiP is formed by D45, D57, H153, and D157. The conserved L152 is a part of a hydrophobic gate between two solvent-filled cavities. *D*, water-mediated diffusion of metal ions in ZIPB.

Zinc transporters are metalloproteins specialized for selective capture and vectorial movements of zinc ions across the membrane barrier. In this two-step molecular process, chemical properties of Zn(II) dictate the composition and geometry of metal-binding sites and their immediate surroundings to afford zinc selectivity. ZnTs and ZIPs are ubiquitous metal efflux and uptake transporters found in bacteria, archaea, and eukaryotes (14, 24, 25). At present, bacterial zinc transporters, YiiP and ZIPB, are two representative ZnT and ZIP homologs for which crystal structures have been determined with bound Zn(II) and/or its isomorphous Cd(II) (Fig. 2, *A* and *B*). The metal selectivities of YiiP and ZIPB have also been explicitly determined, providing the experimental basis for correlating metal selectivity with binding site composition and coordination geometry.

YiiP is an integral membrane protein found in the cytoplasmic membrane of *Escherichia coli* (26). It belongs to the protein family of cation diffusion facilitator (CDF) including mammalian ZnT homologs (27). YiiP was initially thought to be a ferrous iron transporter based on the effects of its deletion or *trans*-expression on bacterial growth and survival (28). However, the presence of redundant metal transport systems and compensatory metal homeostatic controls could lead to misinformation on metal selectivity. A more rigorous determination of metal selectivity was developed using direct measurements of metal uptake into proteoliposomes mediated by purified and reconstituted YiiP proteins. The spectrum of YiiP metal substrates was profiled by inductively coupled plasma–mass spectrometry (ICP-MS), showing that YiiP transported Zn(II) and Cd(II), but rejected all other transition metal ions in the fourth period (29). This selectivity profile for the purified YiiP in the test tube is identical to that of a single-cysteine YiiP mutant in the native *E. coli* plasma membrane (30).

ZIPB is a structural genomic target selected from a large collection of microbial ZIP homologs. It was found in *B. bronchiseptica*, thereby termed ZIPB (31). ICP-MS analysis of ZIPB in reconstituted proteoliposomes provided direct evidence that ZIPB transports Zn(II) and Cd(II), but rejects Fe(II), Cu(II), Co(II), Mn(II), and Ni(II) (31). Thus, ZIPB shares a common metal selectivity with YiiP. Zinc and cadmium are two group-12 *d*-block metals in the fourth and fifth period, respectively. They have similar outer electron configurations but vary in their ionic radii. It appears that common features in the electron configurations of Zn(II) and Cd(II) are exploited to confer Zn(II)/Cd(II) selectivity against transition metal ions with different preference for coordination number and geometry. On the other hand, the accommodation of distinct ionic sizes from 0.74 Å for Zn(II) to 0.97 Å for Cd(II) demonstrates considerable fluidity in size selection, making a critical distinction from the size-based selectivity mechanism used by ion channels to discriminate *s*-block metals, such as potassium and sodium ion (32).

The YiiP crystal structure revealed a Y-shaped dimeric architecture arranged around a twofold axis-oriented perpendicular to the membrane plane (33, 34) (Fig. 2*A*). A cryo-EM structure of YiiP from *S. oneidensis* revealed a distinct inward-facing conformation in the lipid environment (35, 36) (Fig. 2*C*). Each protomer comprises an N-terminal transmembrane domain (TMD) followed by a C-terminal domain (CTD) that protrudes into the cytoplasm. Three distinct zinc-binding sites were found in each protomer. The intramembranous site, also known as the transport site, is localized to the hydrophobic core of TMD and responsible for Zn(II)/Cd(II) selectivity (30). Side chains of four highly conserved residues (D49, D53, H153, and D157) are projected from two antiparallel transmembrane helices (TM2 and TM5) to form a classic tetrahedral Zn(II)/Cd(II)-binding site (Fig. 2*A*). Metal-binding analysis by isothermal titration calorimetry revealed a sub-μM range of Zn(II)/Cd(II)-binding affinity (30, 37). The binding reaction was coupled to the release of 1.23 proton upon each Cd(II) binding, suggesting a 1:1 Cd(II)-for-proton exchange (37). H153 in the tetrahedral transport site is the sole proton-titratable residue under the physiological pH range. This residue may act as a proton donor or acceptor depending on Cd(II) coordination.

The structure of ZIPB showed a monomeric transport unit consisting of eight transmembrane helices (TMs) forming a single helix bundle where the first four TMs (TM1 to TM4) are approximately twofold related to the last four TMs (TM5–TM8) (38) (Fig. 2*B*). These TMs intertwine to embrace a central binuclear metal center within an inner four-helix bundle stabilized by four peripheral TMs and lipid molecules that fill the inter-TM gaps. The binuclear metal center is situated in the hydrophobic core, likely responsible for Zn(II)/Cd(II) selectivity. ZIPB was cocrystallized with Cd(II), and its structure was solved with or without Zn(II) back soaking to partially replace bound Cd(II), yielding two conformations of the binuclear metal center occupied by either two Cd(II) or a Cd(II) and a Zn(II) (38). The metal ions trapped in the binuclear metal center are termed M1 and M2, respectively. They are bridged by one or two carboxylate residues (E181 or E181 + E211) that form bidentate coordination (Fig. 2*B*). M1 is penta-coordinated while M2 is hexa-coordinated. Their coordination spheres are primarily filled by ligands from residues located within two conserved hexapeptides: “^177^HNhPEG^182^” and “^207^QD/NhPEG^212^” (h refers to a hydrophobic residue) in TM4 and TM5, respectively. These TMs are kinked by P180 and P210 to properly place multiple ligating residues. Thus, the binuclear metal center is largely nested between TM4 and TM5, but residues from neighboring TMs, such as M99 from TM2 and E239 from TM6, fill up the coordination sphere. Of note, two coordination sites of M1 and one coordination site of M2 can be occupied by water molecules (38). These coordinated water ligands may exist as hydroxide ions to stabilize two closely associated metal ions within 4.5-Å in a low-dielectric-constant environment of the inner membrane.

The crystal structures of YiiP and ZIPB show that zinc coordination chemistry is richly exploited in different coordination spheres from a classical tetrahedral site to a more complex binuclear site with coordination numbers ranging from 4 to 6. Carboxylate anions of aspartate and glutamate are predominantly employed in the zinc transport sites of YiiP and ZIPB (Fig. 1*C*). The binding of a carboxylate residue offers two alternative binding modes between monodentate and bidentate coordination. A carboxylate can also form coordination bonds with two separate metal ions as a bridging residue. The ability of the carboxylate group to rearrange coordinating ligands gives high flexibility of the zinc coordination sphere while maintaining a constant coordination number (39). As such, Zn(II) and Cd(II) may be accommodated by virtue of their shared preference for common coordination numbers and geometries, despite a large difference in their ionic radii. Although histidine is the most common coordinating residue in zinc enzymes (40), it occurs less frequently in transport sites. Nevertheless, histidine plays a critical role in regulating proton-coupled or pH-dependent zinc transport as either a proton donor or an acceptor. Cysteine is another prevalent coordinating residue in structural sites of many zinc metalloproteins (41); however, it is conspicuously missing in the transport sites of YiiP and ZIPB.

Structure-guided discovery of functional mutations in zinc transport sites provides insights into the roles of individual coordinating residues. The tetrahedral transport site of YiiP is highly conserved, and alanine substitutions to one of the residue quartets resulted in loss of both zinc binding and transport activity in YiiP and mammalian ZnT homologs (30, 42). Thus, individual coordinating residues in the tetrahedral transport site are functionally important. Moreover, YiiP has a DDHD quartet for transporting both Zn(II) and Cd(II), whereas mammalian ZnTs have an HDHD quartet (27), selectively transporting Zn(II) only. A single H-to-D substitution raised the thermodynamic barrier to Cd(II) binding, giving rise to a refined Zn(II) selectivity over Cd(II) in mammalian ZnTs (29). This finding demonstrates tuneability of metal selection by altering the residue composition of the tetrahedral transport site. However, the residue composition of the tetrahedral site is still insufficient to predict metal selectivity of the CDF family members due to potential second shell interactions (41). Other noted variations to the tetrahedral site are composed mainly of Asn and Asp with additional Asn and Asp residues in the immediate surroundings. Such modified transport sites in ZnT10 and a few bacterial homologs are responsible for selective Mn(II) transport (43, 44, 45, 46). The switching of Zn(II) selectivity to Mn(II) is attributed to a preferential Mn(II) binding to Asp and Asn residues in an octahedral coordination geometry (41).

In contrast to essential roles of individual coordinating residues in the tetrahedral transport site of YiiP, individual residues participating in the binuclear metal center in ZIPB are functionally dispensable (47). Comparing multiple ZIPB crystal structures with variations in the binuclear metal center reveals two critical clues. First, M1 coordination is fluidic with alternative coordinating residues and water molecules: H177, E181, Q207, E211, and M99 when M1 is a Cd(II), and E181, Q207, E211, and two water molecules when M1 is a Zn(II) (38). Thus, the loss of a coordinating group by a single alanine substitution could be compensated by the recruitment of an alternative coordinating residue or water molecule. Second, M2 coordination is required for neither zinc transport nor M1 binding (47). Ablation of the M2 site by triple alanine substitutions yielded a mutant crystal structure with no M2 binding, but a nearly identical wild-type ZIPB conformation (47). The absence of M2 binding did not significantly alter the M1 position and its coordination sphere. Moreover, single M1 binding occurs naturally in some ZIP homologs. For example, M2 in mammalian ZIP2 is occupied by a neighboring lysine residue (48).

Functional characterization of the binuclear metal center in mammalian ZIP4 showed that the M1 site is essential to Zn(II) transport, whereas the M2 site is auxiliary (47). The asymmetric functions of M1 and M2 sites reflect clustering of conserved residues around the M1 site while large residue variations around the M2 site may diversify binding properties of the binuclear metal center through M1–M2 interactions to influence the primary transport site at M1 (49). The overall ZIP structure is evolutionally conserved from bacteria to humans, as demonstrated by a close alignment between the bacterial ZIPB crystal structure and a human ZIP4 structural model generated by coevolution-based contact prediction (50). Nearly all ZIP homologs have at least one carboxylate residue in the TM4 and/or TM5 signature motif, allowing two metal ions to be bridged by one or two carboxylate groups. Within this conserved structural framework, variable protein sequences, local structural dynamics, and incoming water molecules are expected to create far more fluidic coordination environments in ZIPs than a single tetrahedral transport site in ZnTs. Existing metal transport data on mammalian ZIPs suggest promiscuous metal selectivity. For example, ZIP8 and ZIP14 are broad-spectrum metal transporters that mediate cellular uptake of Zn(II), Fe(II), Mn(II), and Cd(II) (51, 52). A glutamic acid residue was found in place of a highly conserved histidine residue in the predicted binuclear metal center. This E-to-H substitution may contribute to a shift of metal selectivity away from zinc (53). ZIP4 also exhibits polyspecific binding and transport of Zn(II), Cu(II), and possibly Ni(II) at higher concentrations (54). High-resolution structures of metal–ZIP complexes and accurate experimental profiling of metal substrates are needed to unravel further the structural elements of metal selectivity in mammalian ZIPs.

## Dynamic mechanisms driving zinc mobility

Zinc complexations that obey the 18-electron rule are typically "exchange inert." However, zinc bindings to zinc transporters are directly coupled to translocation across the membrane barrier. The mobility of zinc ions makes a fundamental distinction between zinc transporters and zinc metalloenzymes, where, in the latter, zinc is generally considered as a permanent constituent of catalytic sites (23). Zinc movement in transporter proteins is an energetic process driven by transmembrane electrochemical gradients. A general form of free energy is stored in a proton gradient as an energy reservoir. It drives many secondary transport systems associated with nutrient uptake and maintenance of ionic homeostasis. YiiP transports zinc against its concentration gradient when the membrane becomes energized with a proton gradient. In contrast, ZIPB transports zinc in an opposite direction, down a zinc concentration gradient (31). Hence, distinct mechanisms of zinc transport are expected to couple selective Zn(II) binding to either a proton motive force or a zinc concentration gradient to dislodge bound zinc ions from their coordination spheres to render mobility.

*In-cell* characterization of individual zinc transporters is often hampered by the presence of redundant zinc transport systems, fluctuations of the proton motive force and zinc concentration gradient. A more direct kinetic analysis of zinc transport was developed using purified proteins in proteoliposomes encapsulated with a zinc-sensing fluorescent dye. The initial rate of zinc influx can be measured on a stopped-flow apparatus before the buildup of a significant intraproteoliposome zinc concertation. This *in-test-tube* transport assay allows precise manipulations of proton and zinc gradients while a tight seal of the proteoliposome membrane enables robust measurements of zinc flux with a time resolution down to 5–10 milliseconds. Stopped-flow kinetic analyses of YiiP and a second *E. coli* CDF protein, ZitB, revealed a substrate saturable transport process that can be fitted by the Michaelis–Menten equation (30, 55). This kinetic behavior indicates a two-step process initiated by a Zn(II)/Cd(II) binding followed by a protein conformational change to move the bound metal ion across the membrane. Zn(II)/Cd(II) binding rapidly reaches a steady state while the ensuing conformational change constitutes a rate-limiting step of the transport reaction (30, 55). In an experimental setting with a Cd(II) concentration in equilibrium across the membrane, the stopped-flow application of a proton concentration jump caused a Cd(II) flux in opposition to the imposed proton gradient, whereas depleting protons in the reaction buffer completely stalled Cd(II) transport despite an imposition of a Cd(II) gradient (55). These results clearly demonstrated an obligatory proton-coupled antiport mechanism. The Cd(II)-for-proton exchange stoichiometry was found to be 1:1 by stopped-flow flux analysis, in agreement with the calorimetric titration result as described above (30, 55). Hence, the observed Michaelis–Menten transport kinetics can be explained by a single-site, alternating-access model (56), in agreement with the observation of a single tetrahedral transport site in the crystal structure of YiiP (33, 34).

The intramembranous transport site in YiiP is situated in a hydrophobic environment lacking any pH titratable residue in its immediate surrounding (33, 34). Thus, direct water access to the transport site is expected to act as a proton donor or acceptor to drive an obligatory zinc-for-proton exchange. The zinc-driven proton transport could be probed by microsecond X-ray irradiation to activate water molecules in the zinc translocation pathway where residues in close proximity to the passing water molecules are covalently labeled by hydroxyl radicals and identified by bottom-up mass spectrometry (57, 58). Zinc binding to YiiP was found to trigger a localized, all-or-none change of water accessibility to the transport site and an adjacent hydrophobic gate at residue L152 (59). Millisecond time-resolved dynamics revealed that zinc binding to the transport site drove a rapid TM5 motion coupled to the gating of L152, resulting in alternate exposures of the transport site to two solvent-filled cavities on either side of the membrane. The *in-cell* transmembrane proton gradient of *E. coli* is about one to two pH units around the expected pKa of H153 in the transport site (60). The flipping of H153 as a part of the transport site to either side of the membrane is expected to change its protonation state (Fig. 2*C*). A deprotonated H153 facing a relatively alkaline cytosol would promote zinc binding from the intracellular cavity, whereas a protonated H153 facing a relatively acidic periplasm may facilitate zinc release into the extracellular cavity. As such, an inward pH gradient drives a vectorial zinc efflux in a 1:1 exchange stoichiometry. In this process, protonation and deprotonation of H153 are mediated by water access through the adjacent L152 that is directly coupled to zinc binding to the transport site. Consequently, the gated water access to the transport site enables a stationary proton gradient to facilitate the conversion of the proton potential energy to the kinetic power stroke of a vectorial zinc transport (59).

Contrary to a single tetrahedral transport site and a complete lack of outer shell interactions in YiiP, the crystal structure of ZIPB revealed multiple metal coordination sites characterized by fluidic coordination environments and participations of coordinating water molecules that are stabilized by residues in the second coordination sphere (38). These structural features suggest a transport mechanism distinct from the single-site alternating-access model. Indeed, stopped-flow kinetic analysis of purified ZIPB in proteoliposomes showed that ZIPB-mediated zinc flux is nonsaturable, electrogenic, and voltage-dependent (31). Although ZIPB activity is pH-dependent, ZIPB-mediated zinc transport is not coupled to the proton motive force (31). Instead, the zinc equilibrium potential exhibits a Nernst relationship predicted for the divalent zinc ion while the voltage dependence of the zinc flux also follows the Goldman–Hodgkin–Katz current equation at a symmetrical zinc concentration. These data provide strong evidence that the ZIPB-mediated zinc flux is electrodiffusional through a zinc permeant channel (31). Similarly, human ZIP2 was found to function independent of the proton motive force, but was modulated by the extracellular pH and membrane potential (61). Of note, the zinc flux through ZIPB is extremely slow in comparison with the potassium ion flux through potassium channels. The restricted zinc flow implies highly constrained zinc bindings that limit Zn(II) mobility along a transmembrane conduit. This kinetic behavior is consistent with the binuclear metal bindings in the ZIPB crystal structure. All bacterial ZIPs identified thus far promote zinc influx into the cytoplasm where zinc is buffered to extremely low levels around homeostatic set points (62). From an energetic standpoint, ZIPB may provide a zinc conduit in the membrane barrier that allows zinc to flow into the cytoplasm down its concentration gradient.

The zinc translocation pathway in ZIPB is distinctively different from that of YiiP due to the presence of a sequence of highly constrained zinc ions (38). These bound zinc ions are approximately aligned in a single file and also partially hydrated (Fig. 2*D*). Since ZIPB constitutes a major uptake route for bacterial zinc acquisition, a high zinc-binding affinity is a prerequisite for effective zinc capture (63), but the high affinity may trap bound zinc ions to impede their transmembrane movement. The dynamic process of water access to the binuclear metal center revealed by X-ray footprinting uncovers an active role of hydration water molecules in releasing the trapped zinc ions (64). Contrary to the expectation that zinc binding would block water access as observed in YiiP, multiple zinc bindings in ZIPB were concomitant with increased water accessibility to selective coordinating residues, indicating that zinc ions and water molecules are cotransported (64). In the intramembranous binuclear metal center, zinc coordination fully protected E181. This loss of water access is balanced by increased water access to the Q207-D208-E211 triad and E240 (64). The opposite changes in water accessibility suggest that water entry to subpockets of the binuclear metal center may partially rehydrate the bound zinc ions, switching the mode of coordination residues from zinc binding to release. Mapping water-reactive residues to the ZIPB crystal structure revealed a water translocation pathway that overlapped with a zinc translocation pathway defined by X-ray crystallography (38, 64). Following the intramembranous binuclear metal center, zinc ions navigate through the translocation pathway *via* an interim zinc-binding site to a peripheral binuclear metal center at the cytoplasmic exit (38). These binding sites are closely spaced, allowing a series of ligand exchanges between consecutive binding sites to relay a bound zinc ion from one binding site to another down a zinc concentration gradient (65). The peripheral binuclear metal center is thought to form a high-affinity sink to hold imported zinc before the bound zinc is accepted by cytosolic zinc-binding proteins such as metallochaperones, although zinc-specific metallochaperones have yet to be identified (66, 67). This diffusional mechanism was similarly ascribed to the copper uptake transport Ctr1 where Cu^+^ diffusion is mediated by consecutive Cu^+^-binding sites, leading toward a high-affinity copper sink at the cytoplasmic exit (68).

Comparative structural analyses provide further insights into how protein conformational changes or local structural dynamics achieve differentiated functions of zinc transport in YiiP and ZIPB. Comparing X-ray and cryo-EM structures of YiiP homologs revealed large conformational changes between an inward- and outward-facing conformation, providing direct evidence for the alternating-access mechanism (35). A major conformational change involves pivoting of a four-helix bundle (TM1, TM2, TM4, and TM5) relative to a TM3–TM6 helix pair to flip over the zinc accessibility to either side of the membrane while a minor conformational change involves twisting of TM2 and TM5 in the four-helix bundle (35, 36). Since the transport site is confined exclusively between TM2 and TM5 (34), a small TM2–TM5 shift could lead to a large readjustment of the zinc-coordination geometry in favor of either zinc binding or release, thereby allowing for allosteric regulation of zinc coordination through protein conformational changes (34). As a result, the zinc turnover rate of YiiP is several orders of magnitude faster than zinc exchange rates in typical zinc metalloproteins.

By comparison, no significant conformational change was observed among ZIPB crystal structures when bound metal ions were partially released by soaking crystals in a metal-free buffer or when metal binding to the M2 site of the binuclear metal center was ablated by triple point mutations (47). The findings of partially hydrated Zn(II)/Cd(II) in ZIPB crystal structures provide a critical clue as to the dynamic mechanism driving zinc movements. Multiple crystallographic water molecules were found in the hydration shells of bound metal ions and also in close proximities to protein ligands that participated in binuclear metal coordination. Positional shifts of coordinating residues *via* local conformational dynamics would suffice to switch interactions from a bound metal ion to solvation water molecule, favoring zinc release over binding. This notion is supported by comparative X-ray footprinting analyses of ZIPB with and without zinc binding (64). The reciprocal pattern of water accessibility changes in both binuclear metal centers and their associations with vicinal crystallographic water molecules provide clear structural evidence for direct water access to a sequence of five zinc-binding sites in ZIPB (64). In sharp contrast, a single tetrahedral transport site in YiiP (33) was completely protected from water access by zinc binding (59). Hence, accumulating data support a transport model in which water dynamics complements metal coordination chemistry to confer mobility to trapped zinc ions in ZIPB, highlighting the functional importance of solvated water in driving zinc transmembrane diffusion.

Bacterial YiiP and ZIPB serve as prototypes for mammalian ZnTs and ZIPs, respectively. Detailed structure–function studies lay the foundation for understanding the inner workings of mammalian ZnTs and ZIPs as to how coordination chemistry is built into different protein structures to confer zinc selectivity, but with distinct mechanisms driving zinc mobility. The tetrahedral transport site in YiiP is highly conserved from bacteria to humans. It is likely that mammalian ZnTs harness the proton motive force to actively pump zinc by conformational changes that alternatively expose a single transport site to either side of the membrane surfaces (59). Indeed, a recent cryo-EM structure of human ZnT8 validated a conserved tetrahedral transport site, which is alternately accessible to zinc binding from either side of the membrane during the zinc transport cycle (69). Interestingly, human ZnT8 in the absence of zinc binding to the transport site exhibited heterogeneous dimeric conformations with one protomer in an inward-facing and the other in an outward-facing conformation. The conformational conversion between these two states involved rocking motions of TM1, TM2, TM4, and TM5 relative to the TM3–TM6 helix pair as described for YiiP. The modes of operation in TMD are similar between bacterial YiiP and human ZnT8, but differences were noted in zinc bindings to cytosolic domains. A mammalian-specific HCH motif at the N terminus of ZnT8 contributes to zinc coordination to the CTD of a neighboring subunit. This HCH motif is also connected to the N terminus of TM1, allowing cytosolic zinc binding to influence the stability and transport function of the TMD (69). On the other hand, the binuclear metal center in ZIPB is less conserved and individual coordinating residues are functionally dispensable. This raises a question as to whether a conserved electrodiffusion mechanism of zinc transport exists from bacteria to humans. In bacteria, ZIPB is responsible for zinc uptake as a micronutrient, facilitating passive diffusion of extracellular zinc into the cytoplasm down an inward zinc concentration gradient (7). Likewise, the zinc level in human sera (>10 μM) is many orders of magnitude higher than the homeostatic set point of cytosolic free zinc concentrations in mammalian cells. This imposing zinc gradient is maintained by a multitude of mammalian zinc transporters and intracellular zinc buffering proteins. Up to now, *in-cell* measurements of zinc transport by various mammalian ZIPs have shown a consistent substrate saturable process with Michaelis–Menten steady-state kinetics (38, 48, 50, 61). Such kinetic behaviors seem consistent with a single-site alternating-access model, but more rigorous functional studies of purified proteins in a well-defined experimental setting are required to unequivocally define the transport mechanism for mammalian ZIPs.

## Surfacing of zinc transporters in response to environmental stimuli

While coordination chemistry and protein dynamics dictate selective binding and transport of zinc ions, compartmentalization of zinc transporters in mammalian cells provides the cellular context for the execution of transport functions and their integration into diverse cellular processes. The *in-cell* functions of individual mammalian zinc transporters are determined by their specific homes in various subcellular compartments and their specific molecular partners interlinked within the cellular protein network (interactome). Depending on the type of cells and cellular environments, a zinc transporter could be trafficked to different subcellular destinations and engaged in different protein interactions. This raises two fundamental questions: how a specific zinc transporter is placed in the right subcellular location at the right time in the right amount, and how protein interactions couple zinc transporters to macromolecular machineries to influence cellular processes? At present, there is a paucity of biochemical data on localizations of individual zinc transporters, even less on the organization of the zinc transporter interactome and the dynamics of protein interactions in response to pathophysiological stimuli. We will use relatively well-studied examples of mammalian zinc transporters to illustrate the molecular mechanisms underlying *in-cell* functions. The existing knowledge is far from complete, but the granular data may help define knowledge gaps and take us toward a more complete understanding of the inner workings of mammalian zinc transporters.

We begin with the regulation of zinc transporters on the surface membranes of enterocytes, which are intestinal absorptive cells lining the inner surface of the small intestine. Dietary zinc enters the polarized enterocytes through the apical membrane followed by zinc release into the circulation at the basolateral side. The trans-epithelial zinc movement is orchestrated by coordinated functions of many mammalian zinc transporters, including ZnT1, ZnT2, ZnT4, ZnT5, ZnT6, ZnT7, ZIP4, and ZIP5 (70). Among them, ZIP4 and ZnT1 are a pair of zinc uptake and efflux transporters localized to the apical and basolateral membrane, respectively (71, 72). Their opposite directions of zinc transport and spatial segregation on different surface membranes allow them to play a major role in driving vectorial zinc movement from the intestinal lumen to the blood. Zinc fluxes across apical and basolateral membranes need to be balanced to maintain intracellular zinc homeostasis while the net trans-epithelial zinc flux is also regulated to maintain systemic zinc homeostasis in the face of dietary zinc fluctuations. As a result, the abundance of ZIP4 and ZnT1 on the respective cell surfaces is tightly regulated according to the zinc availability. Zinc deficiency promotes accumulation of ZIP4 on the surface membrane (73, 74, 75, 76, 77). On the other hand, high extracellular zinc levels not only induce internalization of surfaced ZIP4, but also trigger drastic removal of cellular ZIP4 *via* proteasomal and lysosomal degradation pathways (77). Under normal culture conditions, ZIP4 is transcribed and continuously internalized to maintain a low level of cell surface expression (77). Hence, zinc uptake by ZIP4 across the apical membrane is principally modulated by a putative zinc-dependent brake of ZIP4 internalization. When the extracellular zinc level reaches a threshold, auxiliary structural components such as zinc sensors may be triggered to activate ZIP4 endocytosis by releasing the internalization brake.

**Figure 3:**
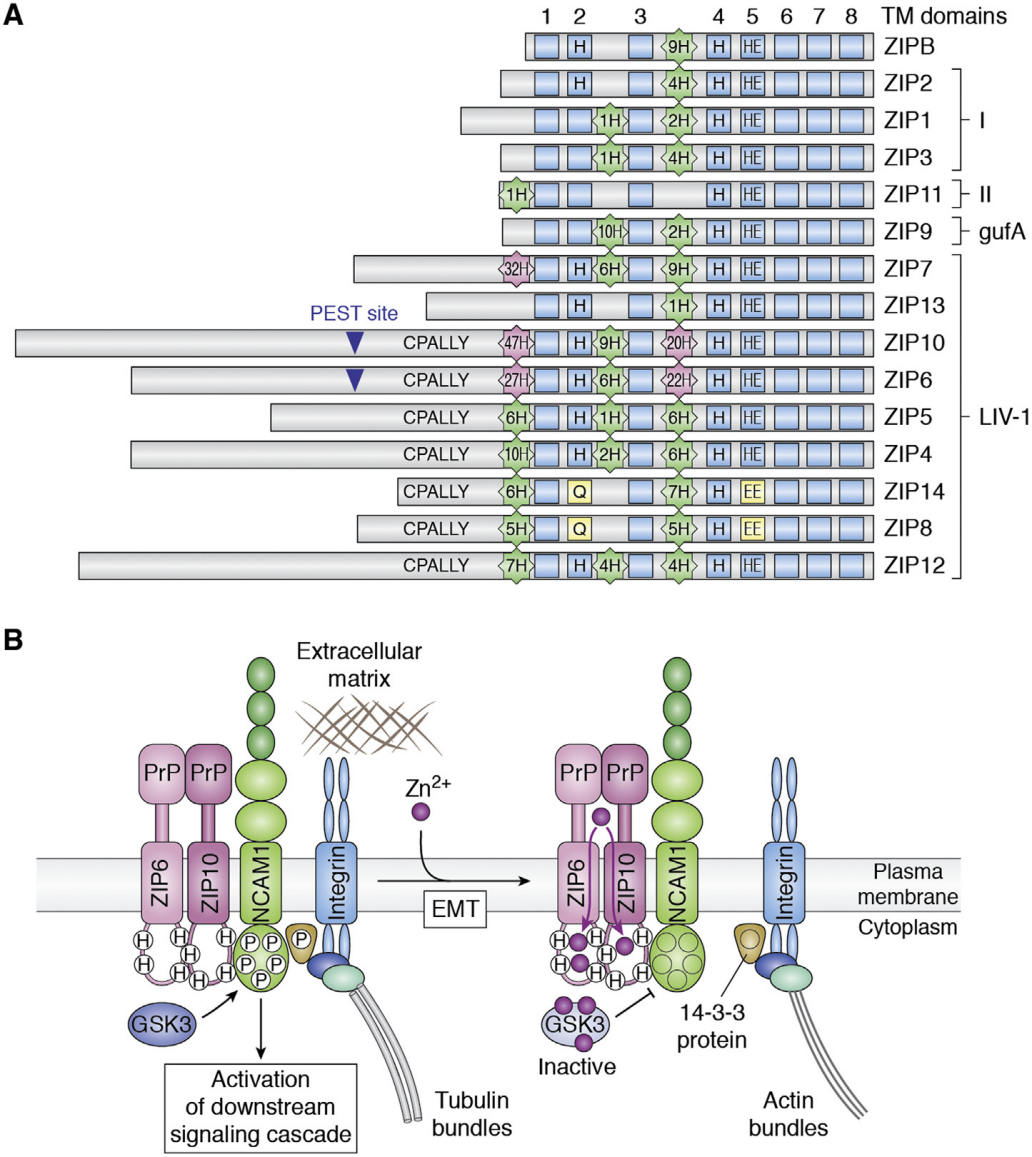
**ZIP-centric interactome**. *A*, topological structures of ZIPs with increased His-abundance in ZIP6 and ZIP10. His residues in the N-terminus, extracellular loop TM2-3 and intracellular loop TM3-4 are shown in *light green* with those greater than 20 are highlighted in *light pink*. TMs are numbered 1–8. The CPALLY motif and the PEST cleavage sites are indicated. Note, conserved His residues in TM2 and TM5 of ZIP14 and ZIP8 are replaced with Q and E, respectively. *B*, zinc-mediated disassembly of focal adhesion complexes. Cell attachment is provided by NCAM1 binding to integrin, which is attached to the extracellular matrix and tubulin bundle when the integrin is phosphorylated by the active form of GSK3. During EMT, a ZIP6–ZIP10 heterodimer in the focal adhesion complex promotes zinc influx, which is captured by the cytosolic His-rich loop between TM3 and TM4 of ZIP6 and ZIP10. An increase of the local zinc concentration inhibits GSK3 (*lighter blue*), resulting in reduced NCAM1 phosphorylation. A reduced phospho-occupancy of NCAM1 sites promotes integrin disassociation from the NCAM1-bound focal adhesion complex, triggering cell detachment. PrP denotes prion protein domain. 14-3-3 proteins are proposed to engage the NCAM1–integrin interaction.

Three potential zinc-sensing elements have been proposed in the human ZIP4 (hZIP4) sequence: a histidine-rich loop (His-rich) in a large N-terminal extracellular domain (ECD), a histidine-containing HxH motif in the extracellular loop between TM2 and TM3, and a His-rich cluster in the cytosolic loop between TM3 and TM4 (Fig. 3*A*). The His-rich loop in the ECD was shown to bind zinc with a low μM affinity (78, 79), and the cytosolic His-rich cluster was shown to bind zinc with nanomolar affinity (80). However, direct evidence for a zinc-sensing role is still missing because H-to-S or G substitution mutations in these sequences caused no changes to zinc sensing of hZIP4 (81). Moreover, intramembranous zinc binding may play a critical role in zinc sensing in hZIP4. D-to-A mutations to coordinating residues in the binuclear metal center abrogated zinc sensing (81). Zinc binding to the binuclear metal center may be allosterically coupled to the cytosolic loop between TM3 and TM4, which is associated with zinc-stimulated endocytosis.

Most ZIPs are localized to the cell surface (52, 82, 83, 84, 85, 86, 87, 88). Thus far, zinc-dependent endocytosis has been reported for ZIP1 and ZIP4. Generally, endocytosis needs specific motifs such as [DE]XXXL[LI] and tyrosine-based endocytosis signals (YXXØ), which operate at the binding site for membrane-bound adaptor proteins (APs) (89, 90). In hZIP4, a [DE]XXXL[LI]-like motif is found in the cytosolic loop between TM3 and TM4, but substitutions of the two Leu residues with Ala residues were insufficient to produce an effect on hZIP4 endocytosis (81). Instead, a conserved LQL motif in the same cytosolic loop acts as a critical determinant for hZIP4 endocytosis (81). The conformational change triggered by zinc binding to the binuclear metal center may induce the LQL motif to fold into a conformation so that hZIP4 is recognizable by yet-to-be identified APs (81). This conformation-dependent endocytosis of hZIP4 is proposed to occur constitutively in a ubiquitin-independent manner under normal conditions with adequate but nonexcessive zinc. Interestingly, all plasma membranes located ZIPs in the LIV-1 subfamily have some forms of the LQL motif, which fits the consensus motif [I,L,S]Q[L,N,D,P,A], suggestive of functional importance throughout the family. In addition to the LQL motif, the aforementioned cytosolic His-rich cluster in the TM3–TM4 loop is involved in zinc-stimulated ubiquitination and degradation of hZIP4 under excess zinc conditions (75). In ZIP1, a [DE]XXXL[LI]-like peptide in the cytosolic loop between TM3 and TM4 mediates ZIP1 endocytosis (91, 92). Because the cytosolic loops are divergent in the protein sequence, the number of His residues, and the length among ZIP proteins (Fig. 3*A*), they may provide unique properties for each ZIP protein, including apical or basolateral sorting of ZIPs, in addition to the regulation of endocytosis.

Zinc sensing and zinc-stimulated endocytosis of ZIP4 are slightly different between the human and mouse homologs. In mZIP4, the HxH motif in the extracellular TM2–TM3 loop is required for a high sensitivity of mZIP4 endocytosis in response to low zinc concentrations (93). However, the HxH motif is not involved in zinc sensing in hZIP4 as discussed above. mZIP4 is constitutively degraded following endocytosis in cells cultured in medium containing low μM zinc concentrations (77). In the small intestine of mouse and rat fed a diet with zinc, ZIP4 is hardly detected (74, 94). Detailed analyses of the mouse–human difference in the kinetics of endocytosis and degradation of ZIP4 are required to gain a more complete understanding of the regulated endocytosis of mammalian ZIP4 and their underlying mechanisms.

ZIP4 surfacing is further regulated posttranslationally. The extracellular N terminus of ZIP4 is proteolytically cleaved during a prolonged zinc deficiency (72, 94). This process is endocytosis-dependent. The cleaved ZIP4, constituting all eight TMs, can also accumulate on the cell surface, thus showing the dispensability of the N terminus for ZIP4 surfacing (72, 79, 93, 94). Nevertheless, the N-terminally cleaved ZIP4 is internalized at a faster rate than the full-length ZIP4, suggesting that the extracellular N-terminal portion may be involved in regulation of the sensitivity to zinc-stimulated endocytosis in addition to zinc transport activity (93). Importantly, a similar N-terminal cleavage has been observed with ZIP6 and ZIP10 (86, 95), which are often involved in trafficking the active form of zinc transporters to the plasma membrane.

In contrast to the surface expression of multiple zinc uptake transporters in the ZIP family, the only zinc efflux transporter predominantly targeted to the plasma membrane is ZnT1 in the mammalian ZnT family (43). Surface expression of ZnT1 is critically important to reducing zinc toxicity by exporting excess cytosolic zinc into the extracellular space (96). The response of zinc-dependent ZnT1 surfacing is opposite to that of ZIP4. Excess zinc exposure would increase ZnT1 surface expression as well as its total cellular expression mediated by transcriptional upregulation under the control of metal response element binding transcription factor 1 (MTF1) (71). Zinc deficiency reduces ZnT1 surface expression, and the internalized ZnT1 is further removed through degradative pathways (71). When translation of ZnT1 was blocked by a protein synthesis inhibitor cycloheximide, ZnT1 was degraded much faster than tubulin under normal culture conditions. As discussed in ZIP4 endocytosis, it is still unclear which APs are involved in ZnT1 endocytosis under zinc deficiency and how high zinc exposure may suspend ZnT1 endocytosis. Furthermore, posttranslational modifications of ZnT1 regulate ZnT1 stability in a zinc-responsive manner. Loss of N-glycosylation at Asn299 in an extracellular loop between TM5 and TM6 stabilized ZnT1, but it did not affect trafficking of ZnT1 to the cell surface. Interestingly, a similar stability control by N-glycosylation was found in ZIP14 (94). N-glycosylation was also required for the iron sensitivity of ZIP14 surface expression (97).

While ZnT1 and ZnT10 are respectively zinc and manganese efflux transporters targeted to the cell surface membrane, mammalian ZnTs generally are zinc-sequestrating transporters localized to intracellular membrane compartments (Fig. 1*A*). Some ZnTs targeted to the secretory vesicles can be transiently trafficked to the surface membrane following exocytotic fusion of secretory vesicles with the surface membrane (98, 99, 100). In pancreatic β-cells, glucose-stimulated insulin secretion is coupled with the surfacing of ZnT8, which may contribute to a cytosolic zinc surge following glucose exposure (99). In mammary gland epithelial cells, lactation hormones trigger surfacing of ZnT2 and ZnT4, the latter may be further facilitated by zinc exposure (101, 102). This type of ZnT surfacing control is operative in secretory cells where a rapid recycling mechanism may be necessary to restore the localization of vesicular ZnTs.

## Functional diversification by multilayers of protein interactions

Protein interactions add another dimension of functional integration to mammalian zinc transporters in addition to subcellular trafficking, posttranslational regulation, and tissue-specific expression. Protein interactions display a continuum of binding strength and stability (103). Many interactions are transient, and alternative interactions may occur in specific cellular contexts or at particular times during cell lineage specification. The dynamic organization of zinc transporter interactomes results in segregation of zinc transporters and functionally related proteins into interconnected protein communities corresponding to multiprotein complexes, subcellular protein colocalizations, and intracellular signaling cascades (104). At the molecular level, individual zinc transporters are assembled into functional modules through homo- and hetero-oligomerization of monomeric ZnT and ZIP homologs. These functional modules are further assembled into protein networks dedicated to specific cellular processes. At present, architectures of zinc transporter interactomes are largely unknown with one exception (see below), but well-studied examples of ZnT/ZIP-interacting proteins provide a glimpse of context-specific organization of zinc transporter interactomes that drive the integration of zinc transport activity to diverse *in-cell* functions.

The first layer of protein interactions takes place among monomeric forms of ZnT and ZIP as the functional unit of zinc transport. Crystal structures of YiiP and ZIPB suggest that extramembranous domains may contain signals directing oligomerization. YiiP is purified as a stable homodimer (105), joined together mostly by two cytosolic CTD with zinc bindings at the dimer interface (Fig. 2*A*) (34). Zinc binding strengthens the CTD–CTD association by neutralizing electronegative surfaces of two opposing CTDs (106). As such, the CTD of YiiP may act as a dimerization joint as well as a cytosolic zinc sensor. Zinc binding to the CTD interface triggers an inter-CTD motion, which alters the coordination geometry of the transmembrane transport site, thereby allosterically regulating its transport activity (34). Human ZnT8 contains two separate tetrahedral zinc-binding sites at the CTD interface in positions similar to that of a binuclear zinc center in YiiP (69). The additional zinc coordinates in ZnT8 are provided by the HCH motif of NTD from a neighboring subunit to establish NTD–CTD interactions to allosterically regulate the TMD conformation as discussed above (69). In addition, dimerization of mammalian ZnTs could be mediated by inter-CTD dityrosine bonds. Covalent dimerization represents a novel mechanism regulating subcellular localization and zinc transport activity of mammalian ZnTs (107).

ZIPB can exist in different oligomeric forms from monomer to homodimer (31, 38) to higher-order polymerization as observed by cryo-EM imaging of purified ZIPB in detergent micelles. The crystal structure of ZIPB was determined in a lipid cubic phase with four outer TMs that create large gaps for lipid filling on the monomer surface (38). Accordingly, delipidation of ZIPB by different detergent treatments influences the nonspecific oligomeric association. Mammalian ZIPs are generally divided into four subfamilies, ZIPI, ZIPII, LIV-1, and gufA (Fig. 1*B*) (15, 107). Among them, members of the LIV-1 subfamily have a large N-terminal ECD, which can independently form homodimers in the absence of any TMs (79, 87). A highly conserved motif, termed CPALLY, is present preceding the TM region in the members of the LIV-1 family that are targeted to the plasma membrane (Fig. 3) (53). This motif consists of the consensus sequence C[X26]CP[X4]Q[X5]C where X is any amino acid (108). Three conserved cysteine residues in this motif are presumably involved in disulphide bonds, which were observed between the first and last cysteine residues in ZIP4 (79). Furthermore, this motif is positioned at the ECD–ECD interface involved in dimerization. Indeed, LIV-1 subfamily ZIPs with the motif, such as ZIP4, ZIP5, ZIP6, and ZIP10, form homodimers (86, 109, 110, 111). Nevertheless, lacking the CPALLY motif did not prevent human ZIP7 and ZIP3 from forming homodimers (112, 113), suggesting that it may play additional roles other than dimerization. All LIV-1 family ZIPs contain multiple His-rich sequences, especially in the ECD and the cytosolic loop between TM 3 and TM4, as demonstrated by ZIP4, ZIP5, ZIP6, ZIP7, and ZIP10, where the total number of histidine residues can exceed 50 (Fig. 3*A*). Zinc binding to the extracellular His-rich cluster was detected at a low μM affinity in ZIP4 as discussed above, while mutations to zinc-binding sites and other conserved ECD sequences could significantly alter zinc transport (78, 79). Hence, the ECD of ZIPs mirrors the CTD of ZnTs in monitoring zinc availability on either side of the membrane to regulate zinc transport through dimerization.

While most ZnTs and ZIPs are thought to be functional as homodimers, heterodimerization occurs naturally among some members of the ZnT and ZIP protein family. A well-characterized heterodimer is ZnT5–ZnT6 (114, 115, 116, 117, 118), which is conserved in eukaryote cells including yeast and plants (119, 120, 121, 122, 123). The functional roles of ZnT5 and ZnT6 in a heterodimer are asymmetrical. ZnT5 contains a canonical tetrahedral zinc-transport site, whereas ZnT6 misses two zinc-coordinating residues by hydrophobic residue substitutions. Thus, ZnT5 is thought to be an operative zinc transporter while ZnT6 a putative auxiliary protomer in the heterodimer (115). ZnT5 also appears to carry the sorting signal directing the ZnT5–ZnT6 heterodimer to the Golgi apparatus (124). ZnT5–ZnT6 heterodimerization is likely mediated by CTD–CTD interactions. This was demonstrated using chimeric ZnT7 whose CTD was swapped with that of ZnT5. The resultant chimera recapitulated ZnT5-like features, that is, formed heterodimers with ZnT6 (115). Moreover, sorting signals in individual ZnT monomers could be combined through heterodimerization to alter subcellular localizations of other ZnT homologs (125). When a pair of fluorescently-tagged ZnT homologs was transiently coexpressed in breast cancer MCF-7 cells, heterodimerization was detected by bimolecular fluorescence complementation (117). The resultant heterodimers were trafficked to altered subcellular locations intended for individual ZnTs (117). For example, ZnT1 was targeted to the plasma membrane in homodimers, but redirected to intracellular vesicles when heterodimerized with ZnT3. In contrast, ZnT3 dimerization was insufficient to redirect ZnT2, ZnT4, and ZnT10 to synaptic vesicles in pheochromocytoma PC12 cells where ZnT3 homodimers were destined to synaptic vesicles (125). On the other hand, ZnT2 and ZnT4 were relocalized to the plasma membrane when heterodimerized with ZnT1 in MCF-7 cells (117), as opposed to their respective homodimer localizations to intracellular compartments (126, 127). These findings revealed a network of ZnT homo- and heterodimers with distinct subcellular localizations, suggesting a potentially prevalent role of heterodimerization in regulating ZnT subcellular distribution. Further studies are needed to validate native ZnT heterodimers and their subcellular localizations in mammalian cells with endogenous ZnT expressions.

Heterodimerization between endogenous ZIP6 and ZIP10 was identified in mouse mammary gland epithelial NMuMG cells based on *in situ* proximity ligation analysis and immunoprecipitation proteomics with tandem mass tag labeling and bottom-up relative mass spectrometric quantification (87). Among mammalian ZIPs, ZIP6 and ZIP10 are the closest paralogues of the LIV-1 subfamily containing an N-terminal prion protein (PrP)-like sequence within the ECDs. ZIP6 and ZIP10 can form respective homodimers targeted to the plasma membrane where they mediate cellular zinc uptake, which often triggers cells to undergo epithelial-to-mesenchymal transition (EMT) (86, 87, 128, 129), a process by which epithelial cells lose their cell–cell adhesion and gain migratory properties to become multipotent mesenchymal stem cells during embryonic development and tissue regeneration. Interestingly, ZIP6–ZIP10 heterodimers can also trigger mitosis, a process of cell division that requires cell rounding to be initiated (130). The full differences between these functional heterodimers are still unclear, as contributions of other ZIP transporters, such as ZIP5, have yet to be clarified. However, accumulating evidence suggests that ZIP6–ZIP10 heterodimerization allows for functional moonlighting as a key regulator in cell morphogenetic programs (87). Since ECDs of mammalian ZIPs are not well conserved or even missing in certain ZIP homologs, heterodimerization among ZIPs is thought to be limited to a subset of ZIPs carrying PrP-like sequences within the ECDs (95, 131). These ZIPs form a separate clade of LIV-1 ZIPs comprising ZIP5, 6, and 10. Heterodimerization among members of other clades of the mammalian ZIP family has yet to be established.

Zinc transporters greatly expand their protein interaction networks through a second layer of protein interactions involving higher-order heterocomplexes with proteins other than zinc transporters. In mammary gland epithelial cells, ZnT2 was found to directly interact with vacuolar proton-ATPase (V-ATPase), and such interactions are critical for V-ATPase assembly associated with secretory vesicle biogenesis, acidification, and secretion (132). In cultured cardiomyocytes, ZnT1 interacted with the β-subunit of the L-type calcium channel (LTCC), inhibiting the trafficking of the LTCC α1-subunit to the surface membrane (133). In epidermal keratinocytes, ZnT1 was shown to form heterocomplexes with EVER1 and EVER2 proteins encoded by genes associated with an inherited skin disorder, epidermodysplasia verruciformis. ZnT1–EVER2 interactions altered the intracellular zinc distribution and inhibited a group of zinc-dependent transcription factors (134). In both cases, ZnT1 was not sorted to its regular destination on the cell surface, but to intracellular compartments by forming heterocomplexes as retention scaffolds (133, 134). In a *Xenopus* oocytes heterologous expression system, ZnT1 and its *C. elegans* homolog CDF1 were found to bind to a regulatory domain of the protein kinase Raf1, facilitating Raf-1 translocation to the plasma membrane where ZnT1–Raf1 interactions activated Raf1, leading to downstream activation of mitogen-activated protein kinase (MAPK) members ERK1 and ERK2 (135). The basal Raf1 activity was inhibited by cytosolic zinc binding, raising the possibilities that ZnT1 upregulated Raf1 either directly *via* protein interactions or indirectly due to zinc efflux in close proximity to Raf1 in a ZnT1–Raf1 complex (135). Hence, accumulating data indicate that heterocomplex formation enables mammalian zinc transporters to perform moonlight functions beyond zinc transport. Finding interaction partners for a zinc transporter in a whole interactome setting may inform on its unconventional functions based on known cellular roles of the associated protein partners.

A mass-spectrometry-based discovery workflow was applied to profiling the ZIP6 interactome followed by global interrogation of its *in-cell* functions using gene ontology (GO) enrichment analysis to assign putative cellular functions and associated processes. As described above, both ZIP6 and ZIP10 are critical players in EMT, but it is unknown which of the ZIP6 interactors links ZIP6 to the cell migratory machinery and how. The ZIP6 interactome in mouse NMuMG cells was captured by anti-ZIP6 co-immunoprecipitation (co-IP) (136). Since the majority of proteins identified in co-IP experiments are nonspecific binders (137), specific ZIP6 binders are shortlisted based on the enrichment of bead-captured proteins derived from wild-type over *ZIP6*-KO cells (138). This quantitative proteomic analysis unequivocally identified ZIP10 as the strongest interactor of the ZIP6 bait (136). ZIP6 was found to interact with ZIP10 but not with other ZIP homologs, indicating an exclusive ZIP6–ZIP10 heterodimeric assembly. Perturbation interactomes and corroborating biochemical analyses further demonstrated reciprocal effects of ZIP6 and ZIP10 on each other's expression and coupled responses of ZIP6 and ZIP10 expression to copper stimulation (136). ZIP6–ZIP10 heterodimerization appeared not to exclude homodimerization in distinct subcellular localizations, because subcellular distributions of ZIP6 and ZIP10 were only partially overlapping in neuroblastoma Neuro2a cells (136). At present, the spatiotemporal dynamics of homo- and hetero-ZIP6 complexes remains to be elucidated.

The second layer of the ZIP6 interactome appeared quite limited: only two additional strong ZIP6 binders were identified in the ZIP6-centric interactome data sets (136). These included calreticulin associated with the ER-resident protein folding machinery and the neural cell adhesion molecule 1 (NCAM1) (136). The latter prompted a second set of NCAM1-centric interactome analyses, establishing prominent interactions of NCAM1 with integrins, a well-established hub protein in focal adhesion complexes that links intracellular actin bundles with the extracellular matrix (ECM) in many cell types to mediate cell anchoring, migration, and ECM signaling (Fig. 3*B*). The overall ZIP6 interactome was much smaller than the NCAM1 interactome and comprised only relatively few interactors not shared by NCAM1 (136). A notable exception was glycogen synthase kinase 3 alpha (GSK3A) and beta (GSK3B) that formed exclusive interactions with ZIP6 (136). The hypothesis-free co-IP proteomics data in combination with bioinformatics and experimental mappings of protein interactions and phosphorylation sites narrowed down the global interrogation of *in-cell* ZIP6 functions to a putative protein hetero-complex: GSK3A/B-(ZIP6–ZIP10)-NCAM1-focal adhesion complexes (Fig. 3*B*). This working model assumes that the (ZIP6–ZIP10)-NCAM1 interactions may be mediated by self-templating heteromerization among PrP-like amino acid sequences within the ECDs of ZIP6, ZIP10, and the ECD of NCAM1, which was shown to bind PrP (139, 140). The GSK3A/B-(ZIP6–ZIP10) interactions are mediated by a cytosolic His-rich loop that establishes a zinc sink at the exiting point of zinc influx as described above (64). Hence, the ZIP6–ZIP10 heterodimer may serve as a scaffold for binding and directing GSK3 kinases to NCAM1 phospho-acceptor sites localized to a cytosolic domain in juxtaposition to the inner face of the plasma membrane. Phosphorylation of these sites was shown to be critical for NCAM1-mediated signaling in astrocytes (141). Since zinc is a highly specific inhibitor of GSK3 (142), an increase of the local zinc concentration by zinc transport through the ZIP6–ZIP10 heterodimer may inhibit the GSK3 activity in close proximity to a ZIP6 heterocomplex, thereby reducing the phospho-occupancy of NCAM1 sites and allowing its dissociation from focal adhesion complexes and the extracellular matrix (Fig. 3*B*). Taken together, profiling ZIP6- and NCAM1-centric interactomes revealed intricate multicomponent protein networking as an example to illustrate how mammalian zinc transporters are engaged in cellular protein machineries and localized to distinct subcellular locations. The positionings of specific zinc transporters in specific subcellular locations and protein complexes provide the molecular basis for precise controls of the local zinc concentration, which would regulate diverse zinc-dependent cellular processes manifested in unique cell biology of specialized mammalian cells. In the following sections, we will discuss how some of the key mammalian zinc transporters are functionally integrated at the cellular level to regulate three important zinc-dependent cellular processes: zinc signaling, unfolded protein responses in the ER, and zinc ectoenzyme activation in the early secretory pathway.

## Cellular zinc signaling

Zinc as a signaling molecule interacts with a multitude of intracellular or extracellular proteins and modulates their activities. While the extracellular zinc concentrations fluctuate with the dietary or serum zinc supplies, transient changes in zinc extracellular concentrations can occur following release of zinc-containing vesicles from neurons, pancreatic β-cells, and secretory cells of the intestine epithelium, mammary gland, and salivary gland. ZnT3 controls vesicular zinc concentrations in presynaptic cells (143), and the released neurosecretory zinc functions intercellularly as a neuromodulator of major postsynaptic channels, thereby modulating both excitatory and inhibitory postsynaptic responses (144). ZnT2 facilitates zinc accumulation into secretory granules of secreting intestinal and mammary epithelial cells (145, 146). In epithelial cells, extracellular zinc regulates the activity of the store-operated calcium channel, forming a cross talk between the extracellular zinc and intracellular calcium signaling (147). In pancreatic β-cells, ZnT8 is highly expressed in the insulin secretory vesicles (148, 149), responsible for zinc enrichment to form zinc–insulin hexamers for crystalline packaging (150). High-glucose exposure stimulates insulin secretion while the cosecreted zinc ions act upon zinc-responsive membrane channels and receptors on the cell surface in a negative feedback loop to inhibit insulin secretion in both autocrine and paracrine fashions (151, 152). The secreted zinc also acts as an endogenous molecular switch that increases insulin delivery to the peripheral target tissues by suppressing hepatic insulin degradation (153). These examples illustrate both short- and long-range signaling roles of extracellular zinc signaling in feedback controls of diverse cellular processes, in which zinc transporters regulate the prestimulation zinc loading in secretory vesicles and activate poststimulation clearance of the localized zinc concentration surge. However, what mediates extracellular zinc signaling is only partially understood. An orphan G-protein coupled receptor belonging to the ghrelin/motilin receptor subfamily, GPR39, has been identified as a metabotropic zinc receptor (ZnR) (154, 155). It was found to sense changes in the extracellular zinc level in pancreatic, gastrointestinal, and neuronal tissues where zinc plays prominent physiological roles (156). GPR39 activities were described in neurons that are postsynaptic to vesicular zinc release and in pancreatic β-cells in which disruption of GPR39 impaired insulin secretion *in vivo* (157, 158). At present, a functional link between GPR39 and mammalian zinc transporters is still unclear. This may be a key to understanding the mechanism by which zinc transporters regulate cellular responses to extracellular zinc fluctuations.

Cytosolic zinc is thought to be an intracellular signal that relays external stimuli to intracellular responses *via* phosphorylation–dephosphorylation (159). In this process, phosphate residues are transferred from one signaling molecule to the next in a consecutive cascade leading to gene expression in the nucleus. Phosphorylation and dephosphorylation are mediated by protein kinases and phosphatases, respectively. Their interplays regulate spatial and temporal signal processing of cell surface stimulations into two general categories of signaling outputs: a graded downstream response that provides feedback to restore cellular homeostasis, and a binary all-or-none response that pushes the cell out of homeostasis, driving cue-based decision-making on cell fates such as proliferation, differentiation, and apoptosis (160). Zinc is an activator of diverse protein kinases (161, 162, 163, 164, 165) and also a reversible inhibitor of phosphatases at physiological concentrations (166). Hence, cytosolic zinc fluctuations could effectively switch on/off phosphor-transfer by synergizing the actions of zinc-dependent protein kinases and phosphatases. In living cells, basal levels of protein kinases and phosphatases undergo constant fluctuations due to stochasticity in gene expression (167). The dual controls of protein kinases and phosphatases by the cytosolic zinc concentration make zinc transporters effective regulators in a role that damps out stochastic fluctuations in noisy phosphor-transferring cascades, thereby improving the fidelity of signal transduction.

Mammalian ZIPs are emerging as important regulators of two modes of phosphor signaling by modulating a graded cytosolic zinc level as well as directly participating in positive feedback loops of the binary decision-making process. Among mammalian ZIPs, ZIP7 is unique for its localization to the early secretory pathway including the ER (168, 169). It functions as a zinc gatekeeper that tightly controls the movement of zinc from the ER zinc store into the cytosol and relies on phosphorylation to activate its zinc transport activity (113). In pancreatic β cells, ZIP7 is the most abundantly expressed among ZIP homologs (170), and its mRNA level is upregulated in response to glucose stimulation (171). Functionally, ZIP7 and the surface-expressed ZIP6 are mutually compensatory in β cells. Double knockdown of ZIP7 and ZIP6 significantly impaired the glucose-stimulated cytosolic zinc surge and insulin secretion (172), suggesting that both ZIP7 and ZIP6 play an important regulatory role in the phosphor transduction from a common extracellular glucose signal diverging to a cytosolic zinc surge and insulin secretion, which is primed in β-cells through activations of both PKC and PKA (173). Zinc is an activator of PKCs as a structural component of a conserved regulatory domain in all PKC isoforms (162). Zinc is also a potentiator of PKA by binding to a zinc-dependent phosphodiesterase to inhibit cAMP hydrolysis (174). The combined transport activities of ZIP6 and ZIP7 may activate PKCs and augment the PKA activity *via* a cytosolic zinc concentration increase (172, 175), promoting glucose stimulated insulin secretion (GSIS).

**Figure 4:**
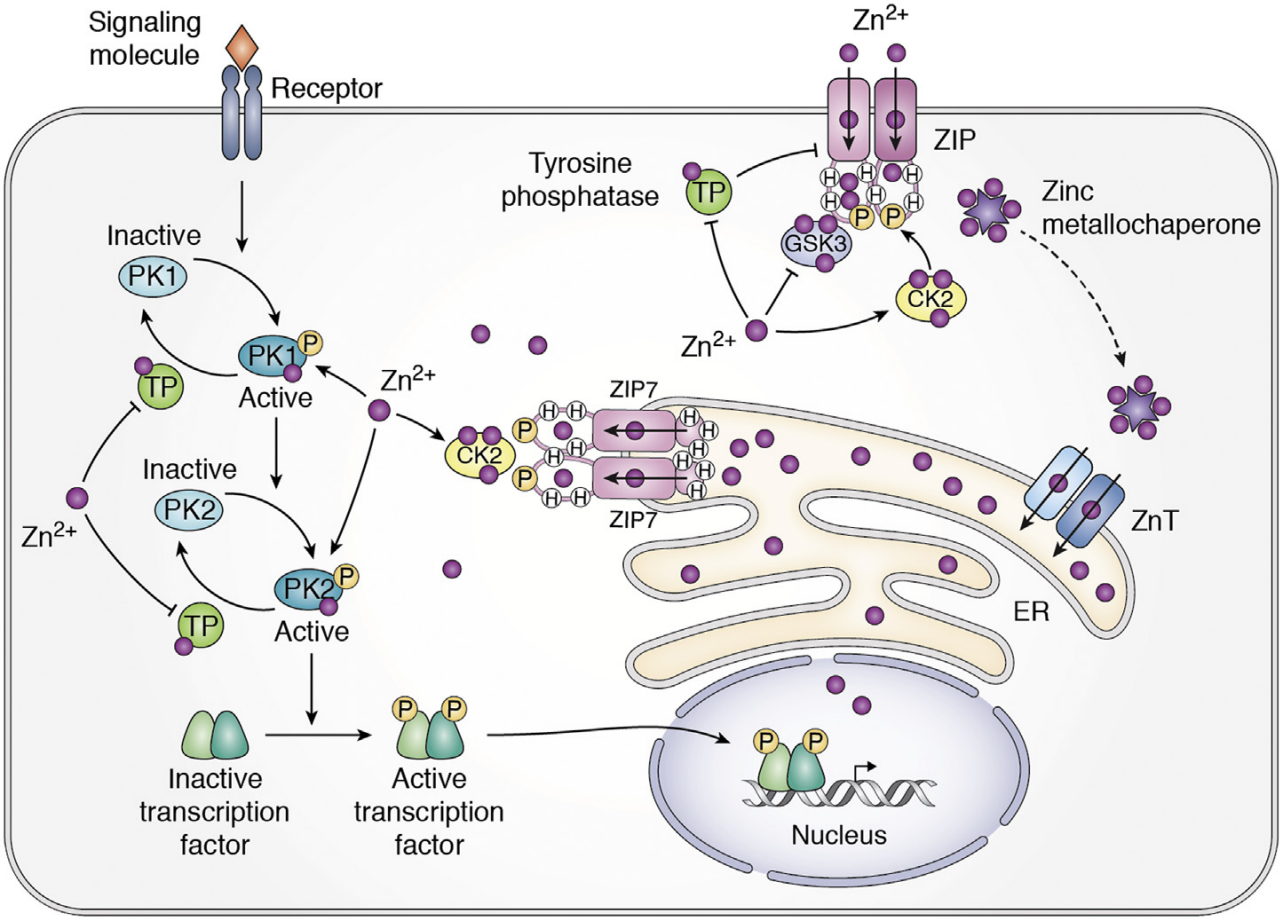
**Zinc regulation of receptor signaling.** Zinc is an activator of diverse protein kinases (PKs) and also a reversible inhibitor of tyrosine phosphatases (TPs). The dual controls of PKs and TPs by the cytosolic zinc concentration improve the fidelity of signal transduction in a phosphorylation cascade leading to transcription factor activation and gene expression. CK2 activates ZIP7 by phosphorylation at Ser residues in the cytosolic His-rich loop, triggering zinc release from the ER store. The increased local zinc concentration upregulates the CK2 activity in a positive feedback loop of ZIP7 activation. Likewise, zinc-dependent PKs, CK2, and GSK3 may activate ZIPs in the plasma membrane to increase zinc influx. Increased local zinc concentrations activate CK2, but inhibit GSK3 to regulate zinc influx in a positive and negative feedback loop, respectively. An increase of the cytosolic zinc concentration could inhibit TPs to shift the balance of ZIP activity to a more activated (phosphorylated) form to augment zinc influx. The depleted ER zinc store may be replenished by joint actions of ZIPs on the cell surface and ZnTs in the ER. A putative zinc metallochaperone is proposed to shuttle cytosolic zinc from ZIP to ZnT to maintain a low cytosolic zinc concentration.

The molecular mechanism linking cell surface stimuli to cytosolic zinc surge and downstream responses was further examined in tamoxifen-resistant MCF-7 breast cancer cells. Extracellular stimuli such as epidermal growth factor plus calcium ionophore elicited a transient protein association between protein kinase CK2 and ZIP7 within minutes (113). ZIP7 phosphorylation on two adjacent residues (S275 and S276) by CK2 triggered ZIP7-mediated zinc release from the ER into the cytosol where an elevated zinc level activated multiple downstream proliferative tyrosine kinase pathways, resulting in aggressive cell proliferation and enhanced migration (Fig. 4). Of note, CK2 is a zinc metalloenzyme containing a zinc finger that mediates dimerization interactions in a multisubunit CK2 complex (176), raising the possibility that zinc itself regulates CK2 activity to complete a feedback loop consisting of CK2-activation of ZIP7, gated zinc release, and zinc-activation of CK2. As such, a positive feedback generates switches for zinc-dependent protein kinases and phosphatases, converting graded phosphorylation responses to a binary cell fate decision on cancer cell proliferation and metastasis in response to environmental cues (Fig. 4).

Tamoxifen-resistant MCF-7 breast cancer cells are characterized by higher endogenous levels of both intracellular zinc and ZIP7, but not other ZIP homologs (108). This unusual cellular attribute suggests a prominent signaling role for ZIP7 as a gatekeeper of intracellularly stored zinc in these cells. Tamoxifen-resistant MCF-7 cells rely on the activation of tyrosine kinases such as epidermal growth factor receptor, IGF1-R, and Src (177) and the downstream activation of MAPK, PI3-K/AKT and mTOR pathways to aid their aggressive behavior (178), which are all driven by ZIP7-mediated zinc release from stores (178, 179). Since ZIP7 and CK2 are ubiquitously expressed, and the CK2 phosphorylation site in ZIP7 is highly conserved in a wide range of different species (113, 168), ZIP7 may function as a general CK2-mediated phosphorylation switch of zinc release from intracellular stores. In cardiomyocytes, high-glucose exposure upregulated ZIP7 expression and induced its phosphorylation to trigger zinc release, resulting in a redistribution of the cellular zinc between the sarco(endo)plasmic reticulum and cytosol (180). In this process, ZIP7 phosphorylation was mediated by CK2, which in turn was upregulated by both high-glucose exposure and elevated cytosolic zinc concentrations, contributing in a positive feedback cycle to further zinc release in response to glucose stimulation (180). In human osteosarcoma MG-63 cells (181), genetic ablation of *ZIP7* resulted in a decreased cytosolic zinc level accompanied by an increased ER zinc concentration. Although ZIP7 deletion induced upregulation of other zinc transporters, these changes were not sufficient to compensate for the loss of ZIP7, suggesting that ZIP7 is obligatory for cytosolic zinc homeostasis and signaling (181).

The phosphorylation sites of ZIP7 were mapped to serine residues localized to the cytoplasmic loop between TM3 and TM4 (182). Among other ZIPs, ZIP6 and ZIP10 have the greatest number of 19 and 20 Ser residues, respectively. A wide variety of kinases including CK2 are predicted to phosphorylate these sites. This observation raises the question of whether there is a link between ZIP7-mediated store release of zinc and the ability of plasma membrane located ZIPs to influx zinc from outside the cell (Fig. 4). Additionally, there are few but noticeable predicted tyrosine phosphorylation sites in ZIP6, ZIP10, and ZIP14. ZIP7-mediated zinc release from zinc stores primarily inhibits tyrosine phosphatases, contributing to a shift of the phosphorylation–dephosphorylation balance toward more phosphorylated forms of ZIP6, ZIP10, and ZIP14. This could potentially activate these ZIPs in the surface membrane to bring in more zinc from the extracellular medium (Fig. 4). Another kinase with numerous predicted sites in ZIPs is GSK3, known to be inhibited by zinc (142). It is tempting to speculate that a possible GSK3–ZIP interaction is mediated by a cytosolic His-rich loop as described above (64). The proximity of this loop to the exiting point of zinc influx may raise the local zinc concentration to inhibit GSK3, thus providing a negative feedback control over zinc transport (80, 182) (Fig. 4). Since ZIP7 has no predicted GSK3 site, the zinc-mediated feedback control may be principally applicable to ZIPs in the surface membrane, particularly to ZIP6 and ZIP10 that contain a zinc sink as suggested by the largest numbers of His-residues in their cytosolic loops (Fig. 3*A*).

In B-lymphocytes, a normal transport activity of ZIP7 is essential for the progression of sequential cellular transitions through quality control checkpoints in an orderly differentiation program (183). Multiple development stages are defined by the recombination status of immunoglobin loci in combination with the expression of cell surface markers (184). Progression of developing B-cells requires sequential integration of multiple signals determined by activities of protein kinases and phosphatases. The formation of the pre-B cell receptor constitutes the first major checkpoint of B-cell development. Hypomorphic mutations of ZIP7 caused a diminished cytosolic zinc concentration, increased phosphatase activity, and decreased phosphorylation of signaling molecules downstream of the pre-B and/or B-cell receptor signaling that impacted the differentiation decision on developing B-cells (183). Importantly, deficiency of ZIP7 alone among many ubiquitously expressed zinc transporters is sufficient to block B-cell development at the pre-B-cell stage (183). These findings highlight a critical role of ZIP7 as a gatekeeper of intracellularly stored zinc in modulating the B-cell receptor signal strength that drives a positive selection of developing B-cells.

While the phosphor-gated transport activity of the ER-residing ZIP7 is required for cell proliferation, differentiation, and normal ER function (181), the ZIP6–ZIP10 heterodimer in the surface membrane may also contribute to regulation of the cytosolic zinc level associated with cell division, especially during G2 and progression to mitosis (185, 186). The essential role of extracellular zinc in cell proliferation was suggested by the ability of zinc to reverse a zinc chelation-induced suppression of cell cycle progression (187). When the cell was ready to progress into mitosis, ZIP6–ZIP10 heterodimer was upregulated on the plasma membrane to increase zinc influx to specifically activate the normal processes of mitosis such as microtubule reorganization and chromosome condensation (130). This mechanism was demonstrated in part by the correlation between antibody inhibition of ZIP6 or ZIP10 and the pause of mitotic initiation, even in the presence of mitotic inducers such as nocodazole (130). These anti-ZIP antibodies are directed to the extracellular epitopes. Antibody binding may physically prevent zinc access or prevent access of proteases to cleave the N-terminal regions, which are required to activate zinc transport. Taken together, accumulating data suggests concerted actions of ZIP6–ZIP10 and ZIP7 in the regulation of cytosolic zinc influxes from both the extracellular medium and intracellular store to control cytosolic signaling *via* zinc-dependent phosphorylation and dephosphorylation processes.

## Regulations of ER homeostasis and unfolded protein response

The ER is a major intracellular zinc store of mobilizable zinc ions that could be released in response to extracellular stimuli as described above (188). The luminal free zinc concentration was estimated to be over 5 nM by an ER-targeted genetically encoded zinc probe, suggesting a large and outwardly orientated zinc concentration gradient across the ER membrane (189). Zinc storage in the ER lumen depends on the buffering capacity of a wealth of nascent zinc-binding proteins and the abundant luminal resident chaperone protein calreticulin, which binds zinc and calcium independently as a multifunctional metal ion sink (190). Interestingly, the major ER zinc-releasing transporter ZIP7 contains a His-rich luminal domain, which may hold on zinc ions and release them into the cytoplasm when activated by phosphorylation (Fig. 4). The ER is also a major organelle responsible for protein folding, maturation, quality control, and trafficking (191). When the ER becomes stressed due to an accumulation of newly synthesized unfolded proteins, the unfolded protein response (UPR) is activated to re-establish normal ER homeostasis. In case of severe and unresolved ER stress, a persistent activation of the UPR triggers apoptosis (192). Zinc is a structural component of many metalloproteins. A lack of zinc when used as a cofactor can adversely affect the metalation of ER-resident proteins, thereby affecting their correct folding and functions. Zinc deficiency in the ER is sensed by the copper zinc superoxide dismutase (SOD1) as a molecular switch for initiating the zinc-dependent UPR (193). Conversely, excess zinc could bind promiscuously in competition against other essential metal ions, yielding misfolded or nonfunctional metalloproteins. Hence, a flux balance between zinc and the nascent unfolded polypeptides is necessary for proper ER functions. As the ER protein burden fluctuates with varying metabolic demands, ER-resident zinc transporters regulate the transmembrane zinc influx to keep pace with changes in the influx of nascent unfolded polypeptides. Among cellular zinc transporters, ZnT5, ZnT6, and ZnT7 are ubiquitously expressed and probably involved in zinc supply into the ER (114, 118, 120), whereas ZIP7 is a major gatekeeper of zinc release from the ER into the cytosol (113). The coordinated actions of ZnTs and ZIPs regulate zinc redistribution between the cytosol and the ER lumen, as such, ER-resident zinc transporters are functional bridges linking the ER zinc load and the protein folding capacity with the cytosolic zinc concentration and zinc signaling.

In an oxidizing environment of ER lumen, free thiols are not available to provide high-affinity zinc-buffering capacity. Fluctuations of the ER zinc concentration with varying cellular metabolic demands may evoke a rapid control over the net zinc flux into or out of the ER lumen. ZIP7 ablation in MG-63 osteosarcoma cells was shown to increase protein disulfide isomerase-A1 (PDI), involved in oxidative folding, and activate the transcription factor C/EBP homologous protein (CHOP) whose target genes increase the ER client protein load and oxidation (194). CHOP activation following the UPR is a hallmark of maladaptation against ER stress that sensitizes cells to ER-stress-induced apoptosis (195). On the other hand, ZIP7 overexpression reduced ER stress with a significant reduction in CHOP and PDI (181). Mice lacking ZIP7 in the intestinal epithelium triggered ER stress in proliferative progenitor cells with a significant increase of apoptotic cell death (196). In human KBM7 myeloid leukemia cells, loss of *ZIP7* resulted in augmented ER stress and impaired tumor necrosis factor receptor trafficking to the surface membrane (197). Emerging data suggests a general protective role of ZIP7 against ER stress. In addition, the surface-expressed ZIP14 was shown to be critical for adaptation to ER stress (198). During ER stress, the UPR-activated transcription factors ATF4 and ATF6α upregulated *ZIP14* expression leading to hepatic zinc uptake. The resultant cytosolic zinc increase was proposed to inhibit protein–tyrosine phosphatase 1B (PTP1B) activity, which acts to suppress apoptosis and steatosis associated with hepatic ER stress. Likewise in human EndoC-βH1 pancreatic β cells, cytokine-induced ER stress also significantly upregulated both *ZIP7* and *ZIP14* expression (170), consistent with protective roles of both ZIP7 and ZIP14 in promoting adaptive UPRs in diverse mammalian cell types.

The requirement of zinc transporters for normal ER functions appears conserved among eukaryotes. A yeast zinc transporter MSC2 is ER-resident and acts as a zinc-sequestrating transporter that delivers cytosolic zinc to the ER lumen. MSC2 is a homolog of mammalian ZnT5 and its zinc-limited mutants exhibit UPR activation and defective ER-associated protein degradation (119). In chicken DT40 lymphoma cells, double deletions of *ZnT5* and *ZnT7* induced UPR due to defective zinc sequestration into the ER lumen (118), suggesting that both excess and deficiency of zinc in the ER could induce ER stress. This is further evidence of how multiple ZnTs and ZIPs work in concert to regulate the balance of ER zinc sequestration and release. Factors that induce ER stress might be alleviated by compensatory regulations of zinc transporter expression and function, whereas maladaptation of zinc transporters exacerbates ER stress and apoptosis.

The coordinated actions of ZnTs and ZIPs in promoting adaptive UPR are further illustrated in the pancreatic β cells, which are professional insulin-producing secretory cells that naturally undergo high levels of ER stress as a result of normal secretory physiology (199). About 50% of biosynthetic capacity in pancreatic β-cells is dedicated to insulin production upon glucose stimulation (200). Glucose stimulation could boost the translation of preproinsulin by ∼50-fold, flooding the ER lumen with nascent peptide chains for folding and the formation of three disulfide bonds per insulin molecule (192). The synthesized insulins are complexed with zinc to form zinc–insulin crystals for storage in insulin secretory granules (ISGs), yielding one of the highest quantities of cellular zinc in the human body (11, 201). ZnT8 is a major zinc-sequestrating transporter responsible for zinc enrichment in ISGs (148, 149) and insulin packaging (150, 202, 203, 204, 205). Accordingly, ZnT8 expression in β-cells is over ten times more abundant than other zinc transporters at the mRNA level (206, 207), and its protein level in EndoC-βH1 cells is compatible to that of the housekeeping α-tubulin (170). Importantly, the subcellular localizations of ZnT8 have an even distribution between the ER and ISGs, contributing significantly to both zinc loading and protein folding in the ER (170).

Surprisingly, the ZnT8 protein level in human EndoC-βH1 cells remained unchanged in the face of large fluctuations of insulin synthesis and secretion upon physiological glucose stimulations (170). In contrast, a trace amount of proinflammatory cytokines triggered a rapid, graded, and reversible degradation of ER-resident ZnT8 while the ISG-resident ZnT8 remained largely unchanged (170). These findings demonstrated exquisite specificity of ER-resident ZnT8 to cytokine stimulations. Transcriptome profiling of cytokine-exposed β cells revealed an adaptive UPR including a marked immunoproteasome activation that degraded ZnT8 and insulin coordinately over a 1000-fold cytokine concentration range. Apparently, the cytokine signaling pathways leading to ZnT8 and insulin degradation are highly intertwined and intersected at a shared ubiquitin proteasome system, highlighting the importance of ZnT8 and insulin decongestion in restoring ER homeostasis under inflammatory insult (170). Among all ZnTs and ZIPs, *ZIP7* is the second highest transcribed gene after *ZnT8* in EndoC-βH1 cells. The reduction of *ZnT8* transcript was functionally synergetic with an increase of *ZIP7* because ZnT8 and ZIP7 transport zinc in opposite directions across the ER membrane (208, 209). In this way, the net zinc influx is reduced to compensate for a reduction of insulin biosynthesis under cytokine-induced ER stress, demonstrating how ER-resident zinc transporters may be coupled with the burden of protein processing to promote ER adaptation to stress conditions (210).

## Activation and trafficking of zinc ectoenzymes in the early secretory pathway

Approximately one-third of all cellular proteins encounter the secretory pathway (211). In yeast, up to 14% of zinc proteins are localized to organelles or extracellularly secreted (212, 213). If this is applied to mammalian cells, more than 4% of total proteins are sorted to be loaded with zinc in the ER and then the Golgi apparatus. Among these proteins, a considerable proportion is thought to constitute zinc ectoenzymes including compartment-resident, membrane-bound, and secretory zinc metalloenzymes. They capture zinc for maturity within the early secretory pathway before trafficking to their final destinations. Loss of zinc in these enzymes could potentially result in misfolding or failure to acquire the proper posttranslational modifications, which would be another trigger of ER stress (118, 119). Zinc metalation in the early secretory pathway is indispensable for some ectoenzymes, because their reduced activities in zinc-deficient cells were not recoverable by a later addition of excess zinc (214). Hence, zinc transport into the early secretory pathway is sophisticatedly maintained from the perspective of functional activation of zinc ectoenzymes.

As described above, ZnT5, ZnT6, and ZnT7 constitute zinc supplying routes to nascent ectoenzymes in the early secretory pathway (114, 118, 215). ZnT5–ZnT6 heterodimers and ZnT7 homodimers contribute to the activation of a number of specific zinc ectoenzymes including ALPs, Ecto-5′-nucleotidase, and Autotaxin (ATX, which is also called ectonucleotide pyrophosphatase/phosphodiesterase 2, ENPP2) (216, 217). However, some ectoenzymes can coordinate zinc at their active site independently of both ZnT dimers (101, 214). Different motif/sequences required for activations of ZnT-dependent ectoenzymes have been identified in mammalian ZnTs and their targeting ectoenzymes. The former example is a unique Pro-Pro (PP) motif present in a luminal (extracellularly) loop between TM3 and TM4 of ZnT5 (actually TM12 and TM13 of ZnT5, because its long N-terminal portion contains extra nine TMs) and ZnT7, which are highly conserved among their yeast and plants orthologs. Substitution of PP with AA significantly reduced ALP activation without affecting their zinc transport activity (218). However, contribution of the PP motif to enzyme activation is less clear in ATX/ENPP2 activation (217), suggesting that certain differences exist in the metalation process of different ectoenzymes. Complementary motif/sequences required for ZnT-dependent ectoenzyme activation was identified by a domain swapping between ATX/ENPP2 and its homolog ENPP3. Both ATX/ENPP2 and ENPP3 require zinc for activity, but their dependency on ZnTs is completely different: the lysophospholipase D activity of ATX/ENPP2 is ZnT-dependent, whereas the adenine nucleotide hydrolysis activity of ENPP3 does not need ZnT-activation. Crystal structures revealed several characteristic differences among members of the enzyme family (219). Uniquely, ATX/ENPP2 lacks a characteristic insertion loop (IL), yet has four residue substitutions in the catalytic domain as compared with ENPP3. The substitution mutations of ATX/ENPP2 resulted in the gain of adenine nucleotide hydrolysis activity but a loss of the ZnT dependency (214). Thus, a fine-tuned zinc metalation mechanism may be explained based on small structural difference on the protein surface, presumably involved in protein interactions.

Some zinc ectoenzymes cannot stably exist in an apo-form in cells lacking both ZnT5-6 and ZnT7 dimers, indicating that they are also closely associated with protein quality control of ectoenzymes. The representative examples include degradation of ALPs through ubiquitin-proteasomal and lysosomal degradation pathways in ZnT5, ZnT6, and ZnT7 triple deficiency cells. This degradative process is irreversible even if excess zinc is added to the cell culture (124, 216). *ZnT5* transcription is induced by ER stress through a transcription factor XBP-1 in HeLa cells, mouse ES cells, and mouse MIN6 pancreatic β cells (118), which is consistent with the notion that ZnT5-6 and ZnT7 contribute to protein quality control in the early secretory pathway as described above. The abilities of ZnT5-6 and ZnT7 to stabilize ALPs may be independent of zinc transport activity, because their transport-null mutants blocked the degradation of ALPs and enabled ALPs to be present as an *apo*-form (216). However, this may be cell-type specific (124). The deletion of the C-terminal glycosylphosphatidylinositol (GPI) anchor, which is attached to the carboxyl terminus of ALPs, made ALPs stabilized in cells lacking both ZnT5-6 and ZnT7 (124). Thus, posttranslational modifications may also contribute to ZnT-dependent stability of ALPs. Further investigation is required to elucidate the molecular mechanisms by which ZnT5-6 and ZnT7 regulate the relationship between activity and stability/degradation of zinc ectoenzymes. Understanding quality control mechanism of zinc ectoenzymes is of pathophysiological importance, because many zinc ectoenzymes are associated with cell fate and activity (211, 220).

ZnT5-6 and ZnT7 also supply zinc to zinc proteins other than zinc ectoenzymes. One of them is the ER chaperone ERp44. Its C-terminal conformation is responsive to zinc binding (221). This zinc-dependent conformational change exposes its substrate-binding surface and the ER retention/retrieval RDEL motif, which drives ER–Golgi retrograde transport of ERp44 accompanied by the capture and retrieval of client proteins such as Ero1α and ERAP1. Although Ero1α and ERAP1 are nonzinc proteins, zinc binding to ERp44 is crucial for their functions in disulfide bond formation, processing, and trafficking of secreted and cell-surface proteins in the ER. Moreover, ZIP7 and possibly other ZIP homologs play pivotal roles in zinc homeostatic control in the ER as described above (196, 222). Intriguingly, both loss and gain of expression of these ZIPs cause no significant effects on stability/degradation of zinc ectoenzymes, arguing against a direct involvement of these mammalian ZIPs in posttranslational modifications of ectoenzyme (124). Apparently, zinc supply to the early secretory pathway may require specific protein interactions to have a direct impact on the metalation and activation of certain zinc ectoenzymes.

## Zinc transporters and human diseases

Clarification of the molecular functions of zinc transporters contributes to the understanding of their causal relationships with major human diseases. An extensive body of preclinical studies using knockout and transgenic animal models has been reviewed elsewhere (223, 224). Here we overview human genetic findings associated with LOF mutations in human ZnTs and ZIPs, their clinical manifestations, and potential mechanism of actions. Thus far, eight types of LOF mutations have been identified and linked to diverse human diseases such as neurodegeneration, immunodeficiency, cancer, and diabetes. Among them, LOF mutations in human ZnT8 are unique for their protection against type-2 diabetes (T2D) (225, 226). All LOF mutations in other human zinc transporters are of deleterious consequences, including zinc deficiencies known as acrodermatitis enteropathica (AE, LOF mutations in *SLC39A4/ZIP4*) (227, 228) or transient neonatal zinc deficiency (TNZD, those in *SLC30A2/ZnT2*) (229, 230, 231), the skeletal and connective tissue disease named spondylocheiro dysplastic form of Ehlers–Danlos syndrome (SCD-EDS, those in *SLC39A13/ZIP13*) (232, 233), agammaglobulinemia (those in *SLC39A7/ZIP7*) (183), Parkinsonism (those in *SLC30A10/ZnT10* or *SLC39A14/ZIP14*) (234, 235, 236), and type II congenital disorders of glycosylation (CDG, those in *SLC39A8/ZIP8*) (237, 238), although the last two diseases are thought to be associated with dysregulations of systemic and cellular manganese homeostasis (Fig. 1*A*).

AE is featured by severe dermatitis caused by malabsorption of zinc in the duodenum and jejunum, attributed to the indispensability of ZIP4 in the process. TNZD is developed in infants exclusively breast-fed by mothers carrying LOF mutations in ZnT2, reflecting an essential role of ZnT2 in zinc secretion into the breast milk in mothers. SCD-EDS is featured by joint hypermobility, skin elasticity, and tissue fragility, and thus ZIP13 plays critical roles for the development of hard and connective tissues. Agammaglobulinemia is caused by LOF mutations in ZIP7, which is required for proper signaling *via* the B-cell antigen receptor (BCR) in early B-cell development as described above. LOF of ZnT10 and ZIP14 develops manganese-induced Parkinsonism (239), attributed to their synergistically biliary manganese excretion: ZIP14 plays a pivotal role in manganese uptake from blood into hepatocytes, whereas ZnT10 have a significant role in manganese excretion into the bile. In contrast, type II CDG is caused by manganese deficiency as a result of impaired ZIP8 activity in reclaiming manganese from the bile. The molecular mechanisms underlying their manganese transport ability are suggested by amino acid differences in the putative metal-binding site within their transmembrane domains, specifically the zinc transport site of ZnT10 (43, 44), or the binuclear metal center in ZIP8 and ZIP14 (240, 241).

In addition to pathogenetic LOF mutations, nonsynonymous single nucleotide polymorphism (SNPs) have been associated with diverse human diseases. For example, rare polymorphic variants impair ZIP4 cleavage, resulting in AE (72). An alanine-to-threonine change at position 391 of ZIP8 resulting from a nonsynonymous SNP (rs13107325C→T) in *SLC39A8* is associated with a variety of diseases including Crohn's disease (242, 243), severe idiopathic scoliosis (244), and circulating lipid levels and risk of coronary artery disease (245). Moreover, aberrant expressions of human zinc transporters are pathogenic. Alterations of ZnT expression have been associated with neurodegenerative diseases including Alzheimer's disease (246) and amyotrophic lateral sclerosis (247). The representatives of ectopic expression are ZIP8 in the cartilage tissue, which causes cartilage destruction (248), and ZIP4 in pancreatic cancer progression, which promotes tumor growth (249).

Similar to ZIP4, a number of ZIPs are upregulated in various cancer cells (108, 179, 250, 251). These often include LIV-1 subfamily members that are present in increased amounts in breast cancer especially ZIP6 (86) and ZIP10 (250), which both encourage EMT and cell detachment involved in metastasis. ZIP7, known to be directly responsible for driving aggressive pathways such as AKT, MAPK, and mTOR (178), is increased in anti-hormone-resistant breast cancer (179). The antibody recognizing phosphorylated ZIP7, the zinc-transporting form (113), is a reliable marker of aggressive cancer (252). ZIP7 is also associated with poor outcome in clinical breast cancer samples (253), consistent with a role in driving these aggressive growth pathways, yet it has also been deemed crucial for enabling the survival of breast cancer patients (254). Since elevated cellular zinc levels generally encourage cells growth and proliferation, a growing variety of cancers has been associated with upregulations of multiple ZIPs. Among them, ZIP7 has been linked to gastric cancer by an ability to drive the AKT and mTOR pathways (255). ZIP8 is one of four genes linked to a risk of developing hepatocellular cancer (256). ZIP10 is one of four genes as a signature of poor survival in glioblastoma (257), and ZIP4 is increased in hepatocellular cancer (258) and likely to increase resistance to treatment in pancreatic cancer by preventing drug uptake (259). Many cancers are driven by tyrosine kinases, and therefore it is not surprising that ZIPs, which generally increase intracellular zinc, are often implicated in increased growth of cancers by zinc-mediated inhibition of tyrosine phosphatases.

*SLC30A8*, the gene encoding ZnT8, was one of the first T2D susceptibility loci identified by GWAS (260, 261, 262). A nonsynonymous SNP in the *SLC30A8/ZnT8* (rs13266634C→T), which causes an arginine-to-tryptophan change at position 325, is associated with increased risk of T2D (260). A major limitation of GWAS is its hypothesis-free approach that tests disease associations without assuming any gene function (263). The next breakthrough in human genetics of T2D was the identification of 12 *SLC30A8* nonsense/frameshift (or LOF) mutations that significantly protect against T2D (226). This large yet unexpected protective effect contradicted a perceived beneficial role of ZnT8 in insulin secretion (264). A causal relationship between ZnT8 activity and T2D risk was established by comparing zinc transport activities of a pair of missense SNP variants (ZnT8-R325W) in reconstituted proteoliposomes (265). The higher-risk variant (R325) was found consistently more active than the lower-risk variant (W325), supporting the notion that reducing ZnT8 activity is beneficial for reducing T2D risk (265). The protective effect of an LOF allele p.Arg138∗ was associated with better insulin secretion due to enhanced glucose responsiveness and proinsulin conversion (266). The p.Arg138∗ allele resulted in reduced ZnT8 expression in human induced pluripotent stem cell (iPSC)-derived β-like cells (266). About 50% of cellular ZnT8 in human insulinoma EndoC-βH1 cells was localized to the ER where ER-associated ZnT8 degradation afforded protection against ER stress induced by proinflammatory cytokines (170). Similarly, downregulation of the cellular ZnT8 level by LOF mutations may constitute a protective mechanism against inflammatory stress, a major pathogenic trigger of T2D (267, 268). Until now, over 150 T2D-susceptibility loci have been identified, confirming the polygenic nature of T2D (269). Many different genetic events can interact together and with environmental factors (270). Only a few of these associated genetic variants are expected to be penetrant enough to cause T2D. A recent whole-exome sequencing analysis identified *SLC30A8* as one of the top four genes that gave the strongest T2D gene-level signals for rare variants, establishing ZnT8 as a top target of prioritization for drug discovery (225).

## Unmet research needs and perspectives

Over the last decades, interdisciplinary research tools have been developed to explore structures, dynamics, subcellular localizations, and protein interactions of individual zinc transporters, and quantitative proteomic studies have begun to elucidate how mammalian zinc transporters are integrated into protein networks. However, major knowledge gaps exist. It is still unclear about the molecular determinant of metal selectivity and the precise transport mechanism of mammalian ZIPs. The hypothetical zinc-specific metallochaperones or other related zinc-trafficking proteins have yet to be identified to uncover the molecular mechanisms driving zinc transfer between individual zinc transporters and their respective zinc donor or acceptor proteins. The molecular identities of membrane-bound APs have yet to be determined to understand their specific interactions with various ZnTs and ZIPs to shuttle between the cell surface membrane and intracellular compartments. Progresses in these aspects will bring clearer answers to the fundamental questions as to where, when, and how zinc is delivered to specific protein targets and subcellular destinations in response to cellular stimuli. At the cellular level, a multitude of ZnTs and ZIPs work in concert to determine the localized zinc concentration dynamics that regulate zinc-dependent cellular processes. The functional interplays and relative contributions of individual zinc transporters to the subcellular zinc distributions and dynamics need to be evaluated globally in the context of competing or compensatory zinc transporters and interacting protein partners. Finally, investigation into *in-cell* zinc transporter functions will be expanded to interrogation of zinc transporter interactomes. The identification of zinc-regulated functional components and their roles in protein networking will provide mechanistic insights into the functional consequences of gain- or loss-of-function mutations in zinc transporters and their impacts on critical cellular processes underlying human diseases.

Existing knowledge of *in-cell* functions of mammalian zinc transporters is largely derived from phenotypic interrogations of gene deletion or overexpression of individual zinc transporters, but a genuine mutation–function relationship may be distorted or even lost if functional compensations by other zinc transporters come into play and stochiometric protein interactions are a determinant factor of the phenotypic outcome. Recent advances in high-resolution mass spectrometry have enabled global profiling of zinc transporter interactomes by co-IP of *endogenous* zinc transporters in complex with their native interacting partners, but complementary tracking reagents and multiplex immunodetection methods are needed to validate proteomic findings by parallel *in-cell* measurements of endogenous zinc transporter levels, posttranslational modifications (*e.g.*, phosphorylation, ubiquitination, and proteolytic cleavage), and protein–protein interactions in a multifactorial space of environmental stimuli over a dynamic concentration range, temporal scale, and subcellular locations of pathophysiologic relevance. Accordingly, the *in-cell* zinc transport functions of individual zinc transporters will be examined in relevant mammalian cells as opposed to the test tube. Pathophysiological stimulations will be applied to perturb cellular hemostasis, and the dynamic process of zinc transporter responses will be monitored to infer their functional roles in restoring homeostasis. While transport activities of individual zinc transporters allow for fine controls of localized zinc concertation gradients in subcellular locations and even in specific protein complexes engaged in cellular processes, these cellular processes are also influenced by a myriad of zinc-independent signals and protein interactions. As such, a pathophysiological stimulation to the cell surface receptor could propagate along parallel signaling cascades and spread through extensive protein networks to yield a complement of input–output relationships, only a fraction of which is dictated or regulated by zinc transporters. These input–output relationships could be monitored in parallel and described in measurable parameters such as stimulation-dependent endogenous protein levels or pairwise protein–protein interactions. Large data sets will be parameterized and fed to machine learning algorithms to unravel inherent interparameter couplings that describe functional integrations of individual zinc transporters into cellular processes in a quantitative and predictable manner. A data-intensive functional description of zinc transporters in native cellular environments represents a paradigm shift from descriptive to quantitative biology of mammalian zinc transporters.

## Conflict of interest

The authors declare that they have no conflicts of interest with the contents of this article.
